# Competence for neural crest induction is controlled by hydrostatic pressure through Yap

**DOI:** 10.1038/s41556-024-01378-y

**Published:** 2024-03-18

**Authors:** Delan N. Alasaadi, Lucas Alvizi, Jonas Hartmann, Namid Stillman, Prachiti Moghe, Takashi Hiiragi, Roberto Mayor

**Affiliations:** 1 Department of Cell and Developmental Biology, University College London, London, UK; 2 Hubrecht Institute, Royal Netherlands Academy of Arts and Sciences, Utrecht, The Netherlands; 3 Collaboration for joint PhD degree between the European Molecular Biology Laboratory (EMBL) and Heidelberg University, Faculty of Biosciences, Heidelberg, Germany; 4 Institute for the Advanced Study of Human Biology, Kyoto University, Kyoto, Japan; 5 Center for Integrative Biology, Faculty of Sciences, Universidad Mayor, Santiago, Chile

**Keywords:** Differentiation, Cell signalling

## Abstract

Embryonic induction is a key mechanism in development that corresponds to an interaction between a signalling and a responding tissue, causing a change in the direction of differentiation by the responding tissue. Considerable progress has been achieved in identifying inductive signals, yet how tissues control their responsiveness to these signals, known as competence, remains poorly understood. While the role of molecular signals in competence has been studied, how tissue mechanics influence competence remains unexplored. Here we investigate the role of hydrostatic pressure in controlling competence in neural crest cells, an embryonic cell population. We show that neural crest competence decreases concomitantly with an increase in the hydrostatic pressure of the blastocoel, an embryonic cavity in contact with the prospective neural crest. By manipulating hydrostatic pressure in vivo, we show that this increase leads to the inhibition of Yap signalling and impairs Wnt activation in the responding tissue, which would be required for neural crest induction. We further show that hydrostatic pressure controls neural crest induction in amphibian and mouse embryos and in human cells, suggesting a conserved mechanism across vertebrates. Our work sets out how tissue mechanics can interplay with signalling pathways to regulate embryonic competence.

## Abstract (web summary)

Alasaadi et al. report the role of hydrostatic pressure in regulating embryonic competence in the developing neural crest.

## Main

Embryonic induction refers to the process by which one group of cells sends a signal to neighbour cells, resulting in a change in the fate of responding cells^1–3^. A pivotal element of embryonic induction is the ability of cells to respond to inductive signals from the inducer tissue, a process called embryonic competence^3–5^, the importance of which has been recognized for over one hundred years^2^. Embryonic competence controls not only the cell types formed during embryonic induction but also the size of the induced tissue and the time at which the tissue can respond to a particular signal. This spatiotemporal role of competence is crucial for patterning tissues and organs during embryo development^1,6^. Embryonic competence not only sheds light on normal developmental processes but also holds promise in informing strategies for new protocols of cell differentiation in vitro, important for regenerative medicine and developmental disorders.

The exploration of genes and signals controlling embryonic competence has thus far resulted in limited success^6–9^, leaving a gap in our comprehension on the mechanisms that control competence. While efforts have been made to understand the molecular basis of embryonic competence, how tissue mechanics influences embryonic competence has remained completely unexplored. In this Article, we investigate the role of mechanical cues in determining competence, leveraging insights from the molecular basis of this inductive process^10–14^. We aim to elucidate the interplay between mechanical and chemical cues in regulating developmental competence. Our result shows that mechanical changes during development control the activity of the mechanotransducer Yap, which in turn is required to modulate the activity of the signalling pathway Wnt, a well-known neural crest inducer. We conclude that neural crest competence is controlled by mechanical cues generated during morphogenesis.

## Results

### Neural crest competence and hydrostatic pressure

**Fig. 1:**
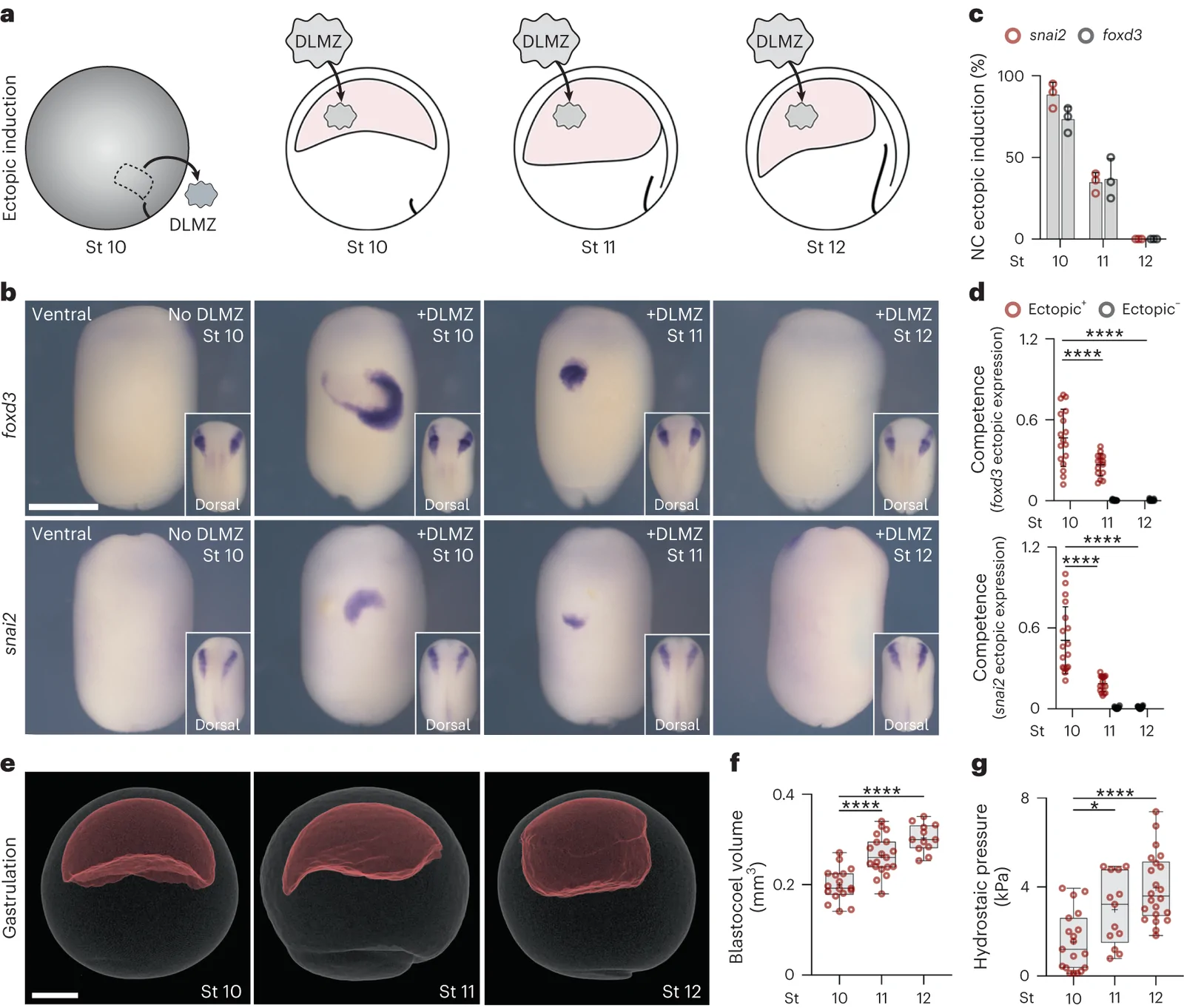
Loss of neural crest competence correlates with increased hydrostatic pressure. **a**, Schematic of neural crest ectopic induction assay using DLMZ as the inducer (grey), grafted into host blastocoel cavity (red). **b**, In situ hybridization analysis of *foxd3* and *snai2* at stage (St) 17 and 18, respectively, seen in ventral view and dorsal view as inset. **c**, Spread of data indicating the percentage of embryos with ectopic induction analysed with different neural crest (NC) markers. **d**, Quantification of neural crest competence at the indicated stages; 10, 11 and 12 normalized to control with no graft. Embryos that exhibited ectopic induction are represented as Ectopic^+^ (red), and embryos with no ectopic induction are shown as Ectopic^−^ (black). **e**, Micro-CT of a whole mount embryo (grey) at stages 10, 11 and 12 showing blastocoel cavity (red). **f**,**g**, Quantification of blastocoel volume (**f**) and hydrostatic pressure (**g**) at stages 10–12. Scale bars, 450 µm for ventral and 200 µm for dorsal (**b**) and 300 µm (**e**). Statistical analysis was performed using two-sided Dunnett’s tests (*P = 0.0164 (**g**), ****P ≤ 0.0001 (**d**,**f**), 95% CI). Data are mean and s.d. (**c**). Box plots (**f**,**g**) show median, 25th and 75th percentiles, and whiskers extending to minimum and maximum values. Three independent experiments (**c**,**d**). *n* = 17_st10_, 19_st11_ and 12_st12_ embryos (**f**) and *n* = 19_st10_, 13_st11_, and 22_st12_ embryos (**g**). Source data

**Extended Data Fig. 1:**
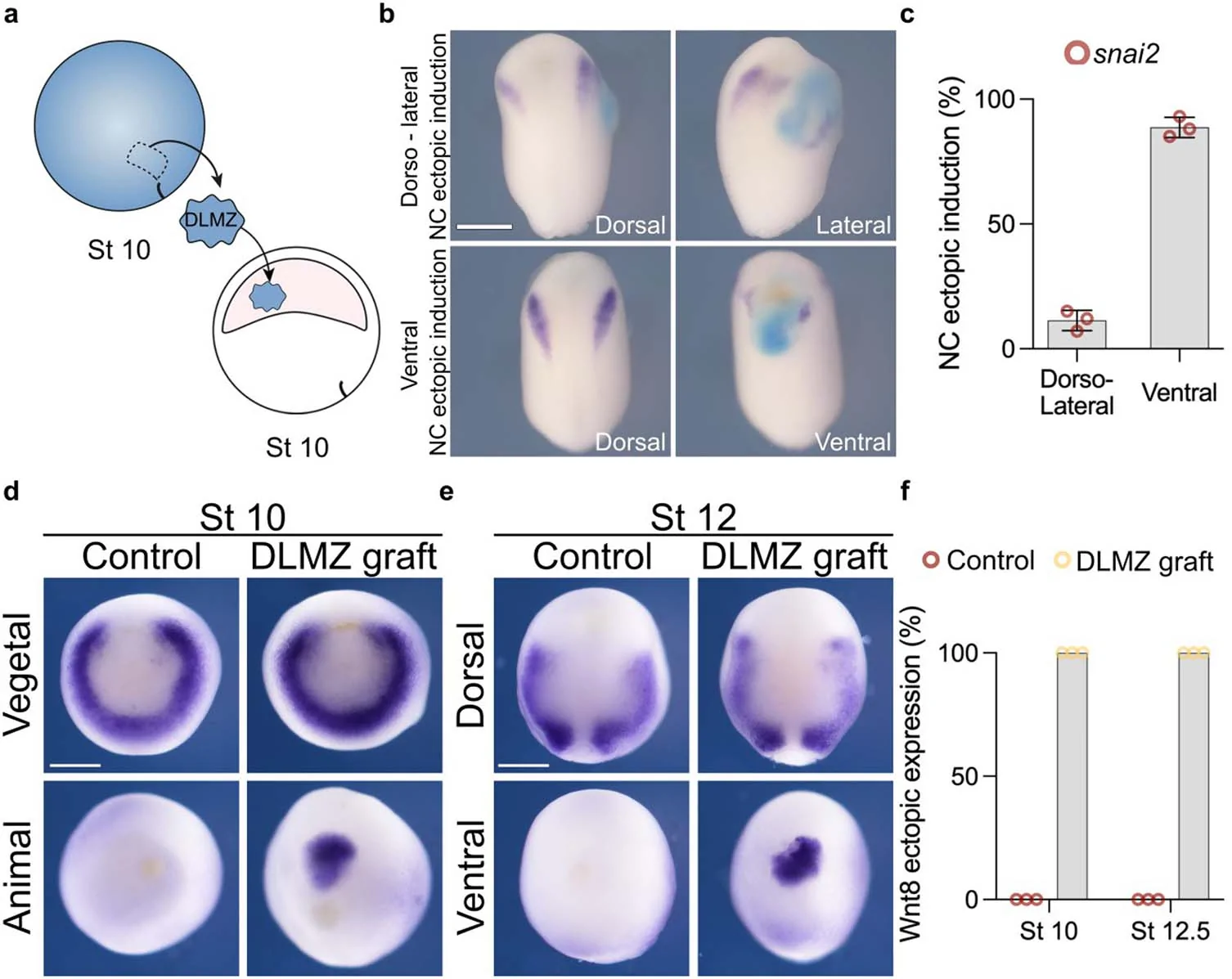
Analysis of neural crest competence. **a**, Schematic of neural crest ectopic induction via DLMZ assay, where stage 10 donor embryo injected with FLDX as a lineage tracer (cyan) in two blastomeres at the 2-cell stage. **b**, In situ hybridization of *snai2* at stage 16, showing embryos with dorso-lateral and ventral ectopic induction. When the grafted tissue was located next to the host neural crest, we observed expansion in the markers, whereas when the graft was ventral, we observed proper ectopic induction; only the latter was considered in the analysis. **c**, Percentage of embryos exhibiting either ventral or dorso-lateral ectopic induction. **d**, In situ hybridization of Wnt8 positive animal and ventral sides after grafting the DLMZ into the blastocoel cavity. **f**, Percentage of embryos exhibiting either positive or negative ectopic Wnt8 expression. Histograms represent mean, error bars are s.d. Panels represent three independent experiments. Scale and 400 µm (**b**) and 350 µm (**d** and **e**). Three independent experiments (**c, f**). Source data

It has been shown that in *Xenopus* embryos a particular mesodermal tissue (the dorsolateral marginal zone, DLMZ) taken from early gastrula (stage 10 N&F)^15^ is a strong neural crest inducer, as it secretes all inductive signals required^10,11,16^. To identify the time at which neural crest competence to respond to inductive signals from the DLMZ is lost in *Xenopus* embryos, DLMZ were grafted into the blastocoel of successively older embryos (Fig. 1a; stages 10–12, Extended Data Fig. 1a–c), cultured until mid-neurula, followed by analysing the expression of the neural crest marker *snai2* (ref. ^10^) and *foxd3* (ref. ^17^). Our results show that ectopic induction of neural crest in vivo is strongest at stage 10, intermediate at stage 11 and lost at stage 12 (Fig. 1b–d), as previously described in vitro^11^. We investigated whether the decline in neural crest induction by DLMZ from stages 10 to 12 resulted from reduced *Wnt8* expression—the molecule known for inducing the neural crest^13,14,16^—when DLMZ was exposed to the blastocoel cavity. We examined *Wnt8* expression post-grafting to determine if any alterations occurred. However, no variations were observed in *Wnt8* expression levels within DLMZ after grafting it into the blastocoel of stage 10 or 12 embryos (Extended Data Fig. 1d–f). This suggests that the diminishing neural crest induction is not caused by changes in the inducer molecule secreted by the DLMZ but rather by a loss of competence in the responding cells.

**Extended Data Fig. 2:**
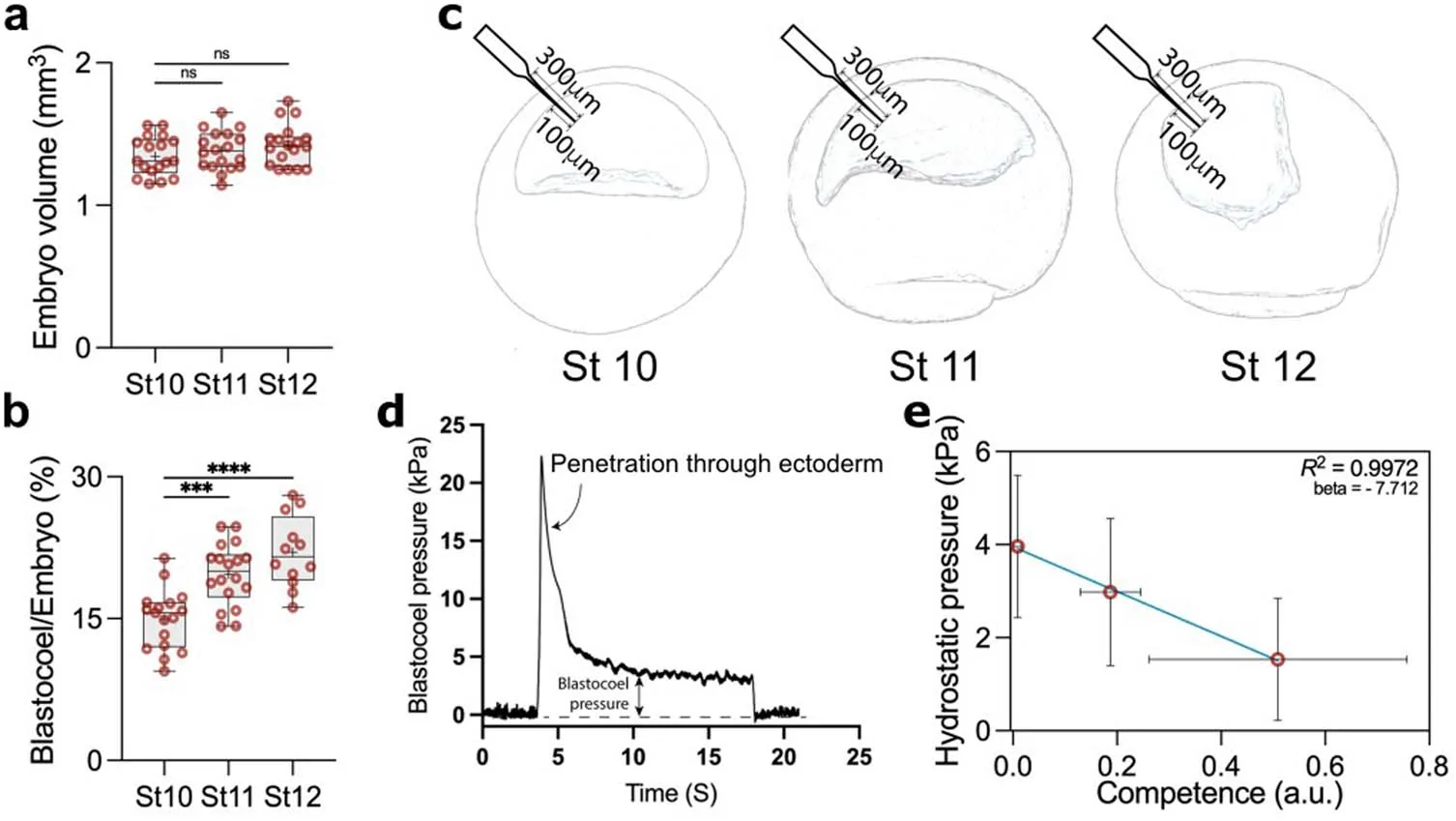
Changes in blastocoel volume and hydrostatic pressure. **a**, Quantification of embryo volume at stage 10 (*n* = 18 embryos), 11 (*n* = 18 embryos), and 12 (*n* = 18 embryos). **b**, Percentage of the ratio of blastocoel volume to whole embryo volume at stage 10 (*n* = 17), 11 (*n* = 18), and 12 (*n* = 12). **c**, Schematic of embryos at different stages showing microelectrode penetration (0.5-1 µm tip) through ectoderm. **d**, Profile of a successful measurement of blastocoel hydrostatic pressure. Pressure is close to zero whilst the microelectrode is not in contact with the embryo before and after measurement. A transitory spike is noted during penetration of the microelectrode through the ectoderm. The spike is followed by a stable phase between five to ten seconds, indicating the actual blastocoel pressure. **e**, Correlation between Hydrostatic Pressure and Competence. ‘R^2^ = 0.99 and beta = −7.7, as calculated using orthogonal distance regression (ODR) to account for measurement variability in estimating neural crest competence and the hydrostatic pressure within the blastocoel cavity. Statistical analysis was performed using two-sided Dunnett’s test (NS, *P* > 0.1639; **a**, ***P = 0.0003; **b**, ****P ≤ 0.0001; **b**, CI = 95%). Box plots (**a** and **b**) show median, 25^th^ and 75^th^ percentiles, and whiskers extend to minimum and maximum values. Data represent mean, error bars are s.d. (**e**). Three independent experiments (**e**). Source data

We noticed that loss of competence during this stage is accompanied by a change in the size of the blastocoel cavity^18^, which we decided to explore further. To precisely determine the change in blastocoel volume, we performed micro-computed tomography (micro-CT) scans of embryos at different stages (Fig. 1e). An increase in blastocoel volume was observed that correlated with the loss of competence (Fig. 1f and Extended Data Fig. 2a,b), which led us to hypothesize that physical changes in the blastocoel might be related to the loss of neural crest competence. As it is unlikely that cells sense the volume of the adjacent cavity directly, we investigated other physical properties that might depend on blastocoel fluid, including hydrostatic pressure, as a promising candidate. Using a micro-pressure probe^19^, we measured the hydrostatic pressure of the blastocoel cavity (Extended Data Fig. 2c,d), and we found a marked increase in the hydrostatic pressure between stages 10 and 12 (Fig. 1g). These stages correspond to when the ectoderm loses its competence to generate neural crest in response to a DLMZ graft, showing a correlation between hydrostatic pressure and competence (Extended Data Fig. 2e).

### Hydrostatic pressure controls neural crest competence

**Fig. 2:**
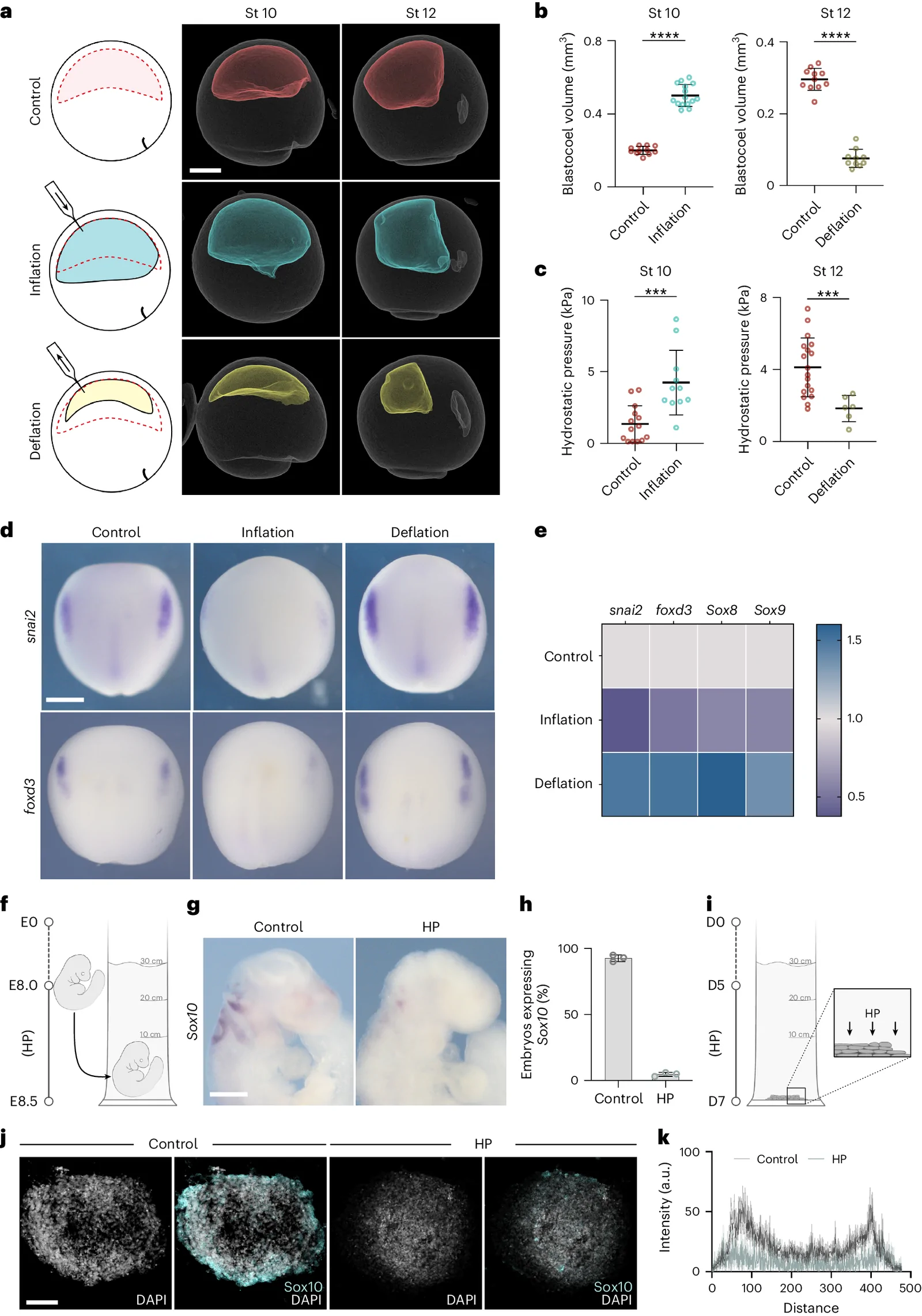
Loss of neural crest induction is driven by an increase in hydrostatic pressure. **a**, The mechanical assays (left) and micro-CT scans (middle and right) of control (red), inflation (cyan) and deflation (yellow) embryos. **b**,**c**, Quantification of blastocoel volume (**b**) and hydrostatic pressure (HP) (**c**). **d**, In situ hybridization (ISH) of *snai2* and *foxd3* at stage 15 after the indicated treatments. **e**, Relative expression of neural crest markers by RT–qPCR at indicated treatments. **f**, Schematic of mice hydrostatic pressure assay, in which mouse embryos are collected at embryonic day 8 (E8) and incubated to E8.5. **g**,**h**, ISH of *Sox10* of mouse embryos (**g**) and percentage of embryos positive for *Sox10* (**h**). **i**, Schematic of neuruloids hydrostatic pressure assay, in which neuruloids were incubated under hydrostatic pressure from day 5 of the protocol until day 7. **j**, Immunofluorescence of neuruloids at day 7 stained with the Sox10 neural crest marker and nuclei counterstained with DAPI. **k**, Quantification of neuruloids culture. Scale bars, 300 µm (**a**), 400 µm (**d** and **g**) and 100 µm (**j**). Data are mean and s.d. Statistical analysis was performed using unpaired two-tailed unpaired *t*-tests (***P ≤ 0.0005 (**c**); ****P ≤ 0.0001 (**b**), 95% CI). *n*_st10_ = 11_control_, 15_inflation_ and *n*_st12_ = 11_control_, 10_deflation_ embryos (**b**), *n*_st10_ = 14_control_, 11_inflation_ and *n*_st12_ = 18_control_, 16_deflation_ embryos (**c**), and three independent experiments (**d**–**f**,**h**,**j**). Source data

**Extended Data Fig. 3:**
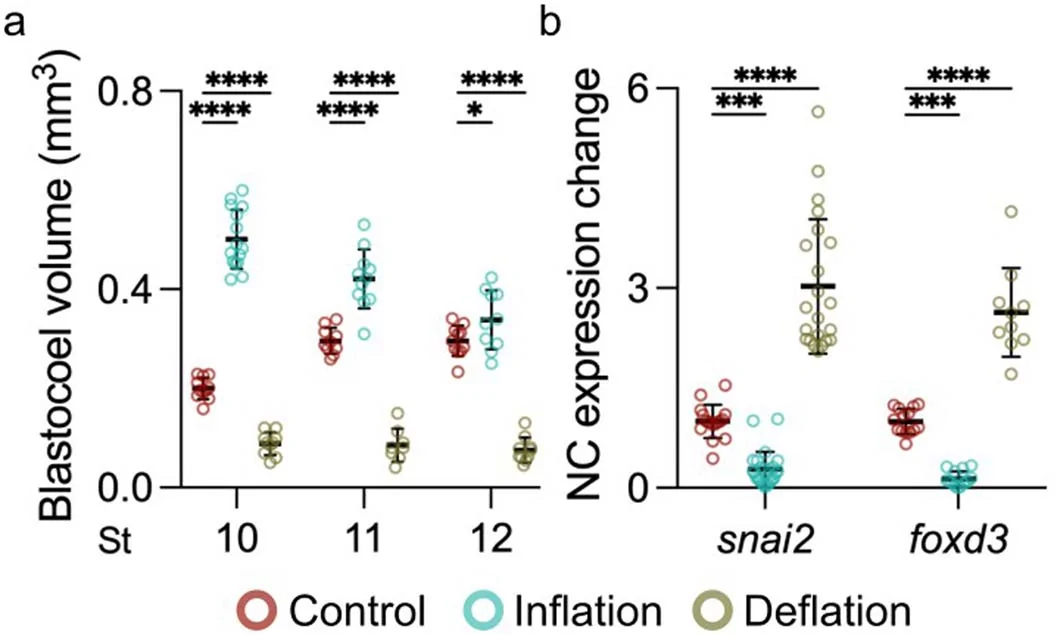
Manipulation of blastocoel volume during gastrulation. **a**, Spread of data points indicating change in volume between control, inflation, and deflation at stage 10 (*n* = 11, 15, 10 embryos, respectively), 11 (*n* = 10, 11, 8 embryos, respectively), and 12 (*n* = 11, 10, 10 embryos, respectively). **b**, Quantifying *snai2* and *foxd3* expression levels by ISH after indicated treatment. Data represent mean, error bars are s.d. Statistical analysis was performed using two-sided Dunnett’s test (*P = 0.0106; **a**, ***P = 0.0001; **b**, ****P ≤ 0.0001; **a** and **b**, CI = 95%). *n*_*snai2*_ = 22_control_, 25_inflation_, 22_deflation_, *n*_*foxd3*_ *=* 22_control_, 14_inflation_, 10_deflation_ embryos (**b**). Source data

**Extended Data Fig. 4:**
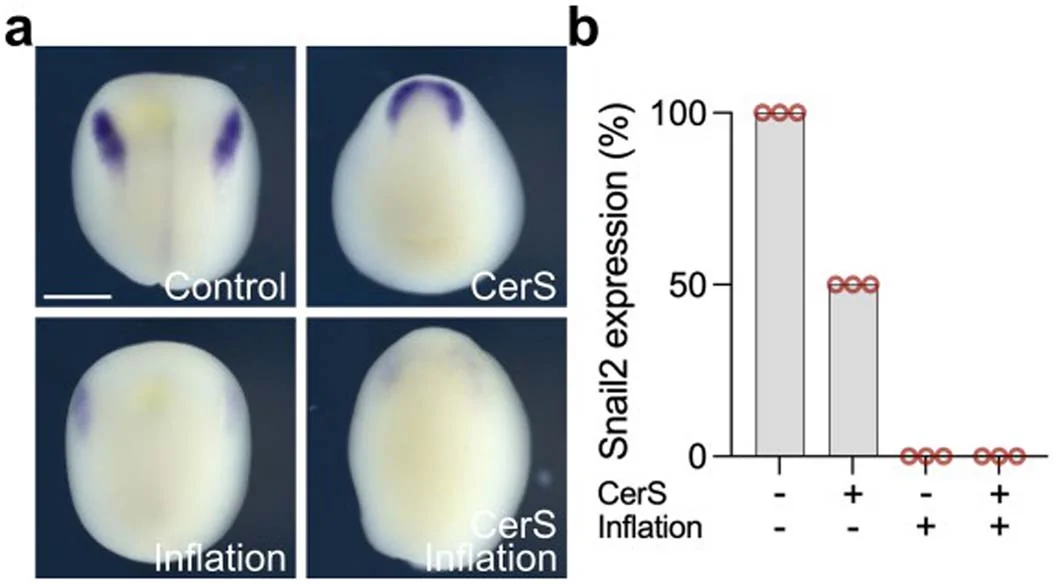
Mesoderm independent neural crest induction inhibition by hydrostatic pressure. **a**, In situ hybridization of *snai2* at different treatments. **b**, Spread of data indicating *snai2* expression percentage. Scale and 400 µm (**a**). Three independent experiments (**b**). Source data

To examine the causal link between hydrostatic pressure and neural crest induction, we directly altered the hydrostatic pressure by increasing (inflation) or decreasing (deflation) the blastocoel volume (Fig. 2a). Inflation was performed by injecting the normal culture medium into the blastocoel cavity, which increased its volume (Fig. 2b, stage 10) and hydrostatic pressure (Fig. 2c, stage 10), whereas deflation was performed by aspirating liquid from the blastocoel cavity leading to embryos with reduced blastocoel volume (Fig. 2b, stage 12) and hydrostatic pressure (Fig. 2c, stage 12). The same treatment was performed across different stages (Extended Data Fig. 3a). Inflation of the blastocoel cavity showed inhibition of neural crest markers *snai2* and *foxd3*, analysed by in situ hybridization (Fig. 2d and Extended Data Fig. 3b) and *snai2*, *foxd3*, *sox8* and *sox9* by quantitative PCR (qPCR) (Fig. 2e), whereas deflation showed an expansion in the expression domains of those genes. These observations are consistent with our hypothesis that hydrostatic pressure regulates neural crest induction, suggesting that mechanics could control neural crest competence to induction by DLMZ. However, as it has been shown that neural crest can also be induced independently of the DLMZ^20^, we tested whether this DLMZ independent induction was also affected by hydrostatic pressure. Mesoderm, including DLMZ, was inhibited by expressing the nodal inhibitor Cerberus-short, as previously published^20^, followed by inflation of the blastocoel and analysis of neural crest markers. Although mesoderm inhibition reduced neural crest induction, the neural crest makers were still visible in the Cerberus-short-expressing embryos; however, blastocoel inflation led to a similarly strong inhibition of neural crest in control and in mesoderm-lacking embryos (Extended Data Fig. 4). This indicates that hydrostatic pressure controls neural crest competence regardless of the inducer tissue. Next, we asked whether this role of hydrostatic pressure is conserved across species. We applied exogenous hydrostatic pressure to mouse embryos (Fig. 2f–h) and to neuruloids derived from human induced pluripotent stem (hiPS) cells (Fig. 2i–k), observing an inhibition in neural crest induction in both systems, similar to our result in *Xenopus* embryos, indicating a widespread role of hydrostatic pressure on neural crest induction. As *Xenopus* embryos are more amenable to in vivo manipulations than mouse embryos, we decided to focus our work on *Xenopus* to further examine the role of hydrostatic pressure on neural crest induction.

### Control experiments for hydrostatic pressure manipulations

**Extended Data Fig. 5:**
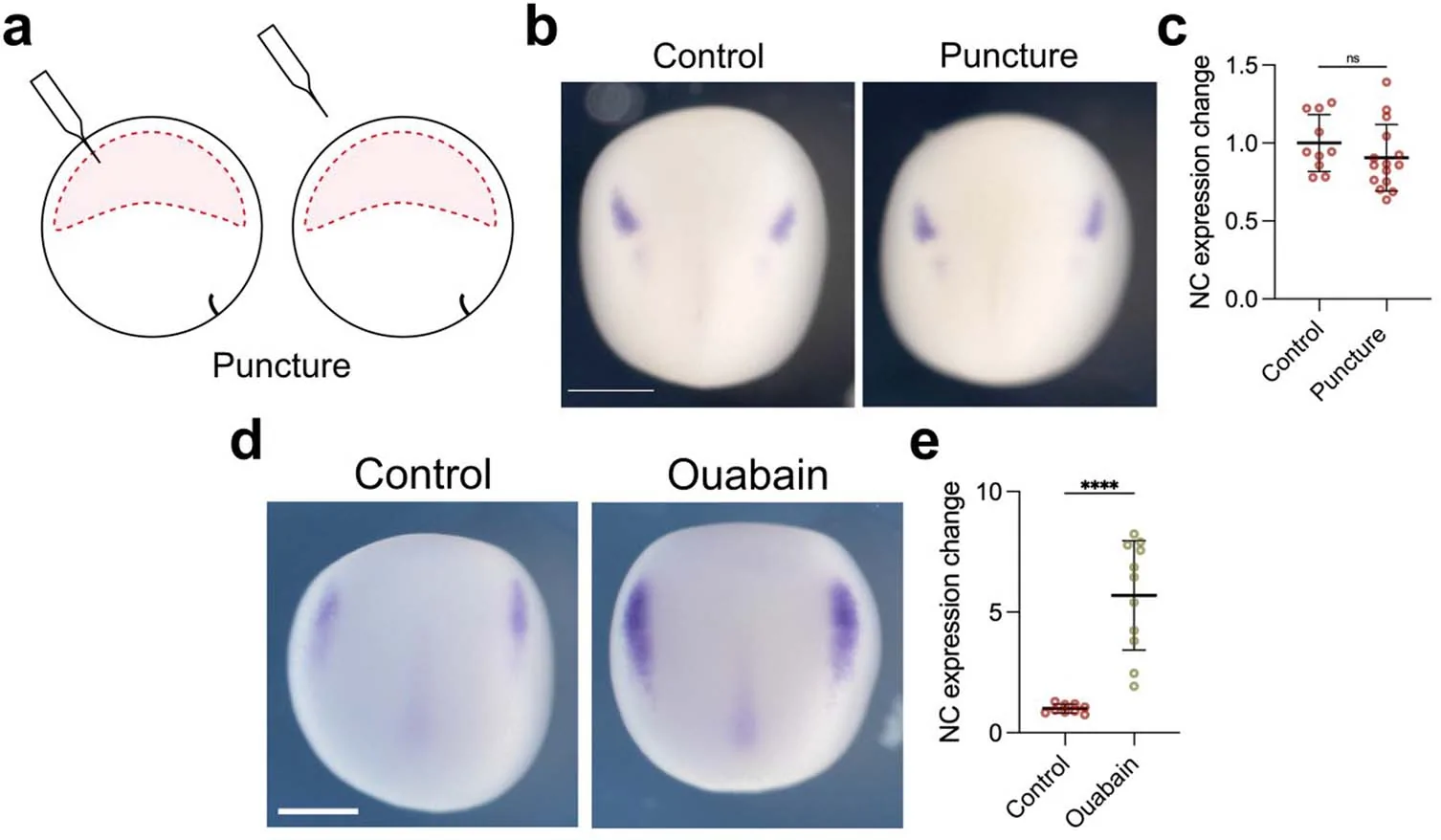
Control for mechanical manipulation of the blastocoel. **a**, Schematic showing the puncture procedure of inflation and deflation. Embryos were punctured with a needle similar to the one used for the inflation/deflation experiments, but no liquid was injected or removed from the blastocoel cavity. **b**, Embryos analyzed via in situ hybridization at stage 15 using *snai2* marker. **c**, Spread of data points indicating the change in neural crest expression in control embryo (*n* = 10 embryos) and punctured embryo (*n* = 15 embryos). **d**, Control embryo compared to embryo treated with ouabain as described in Methods, analyzed at stage 14 using *snai2* marker. **e**, Spread of data points comparing change in neural crest expression between control embryo (*n* = 9 embryos) and treated embryo (*n* = 11 embryos). Scale bar 450 µm (**b** and **d**). Data represent mean, error bars are s.d. Statistical analysis was performed using a two-sided unpaired student *t*-test (NS, *P* = 0.2667; **c** and ****P ≤ 0.0001; **e**, CI = 95%). Source data

As inflation and deflation require a perforation in the embryo, we showed that a similar perforation with an equivalent needle but without inflation or deflation did not affect neural crest induction (Extended Data Fig. 5a–c), indicating that inserting a needle in the ectoderm does not affect induction. To further confirm the notion that blastocoel volume affects neural crest induction, we modified its volume using a different method. The volume of the *Xenopus* blastocoel is maintained by the activity of the Na^+^,K^+^-ATPase^21^, whose pharmacological inhibition via ouabain results in a decrease in blastocoel volume^21^. Embryos treated with ouabain showed an expansion of the neural crest domain (Extended Data Fig. 5d,e), similar to the one produced by our deflation experiments (Fig. 2d,e). Thus, our mechanical (deflation) and pharmaceutical (ouabain) treatments elicit neural crest expansion, suggesting specificity in our treatment.

**Fig. 3:**
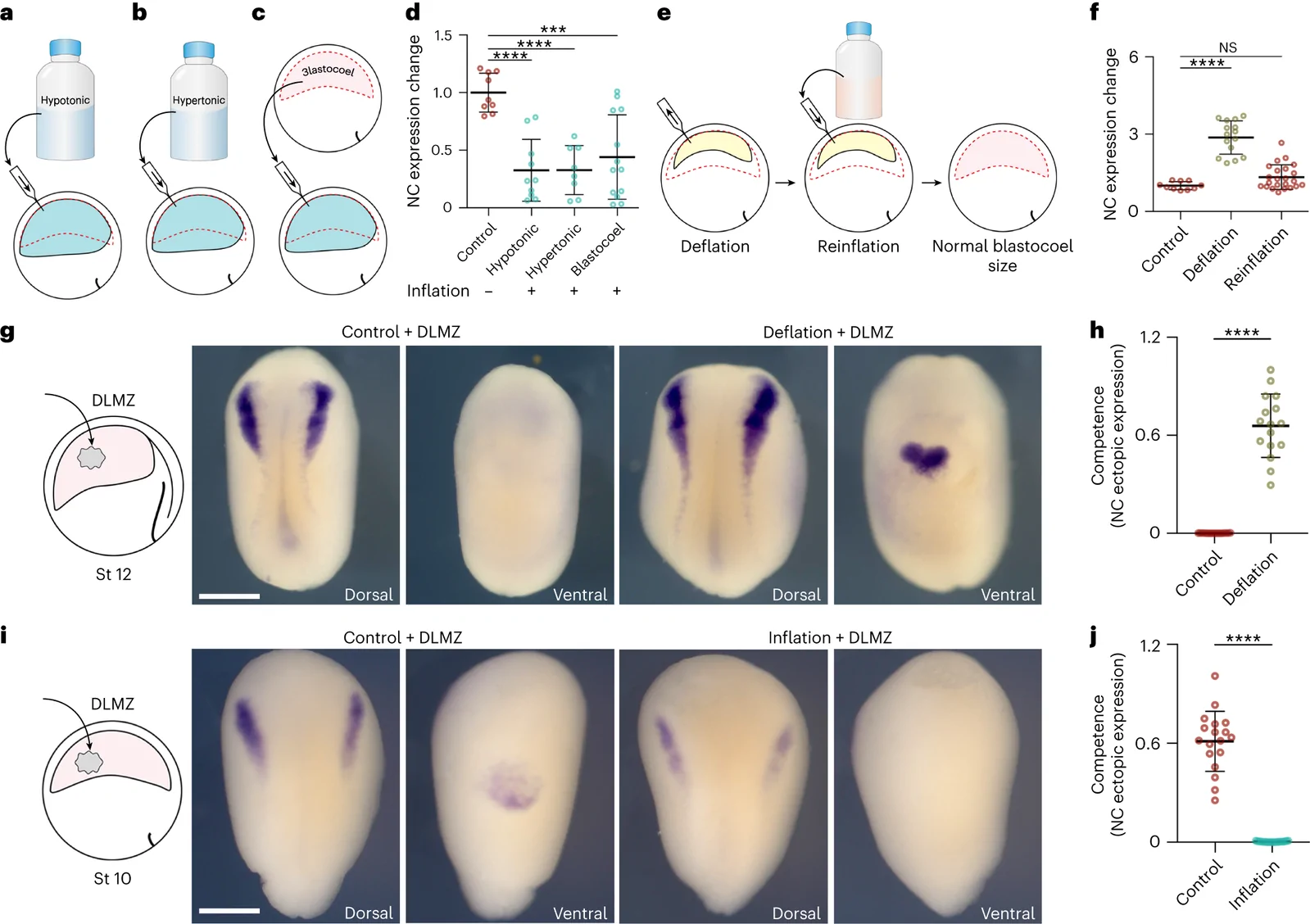
Hydrostatic pressure controls neural crest competence. **a**–**c**, Schematic of inflating embryo with hypotonic (**a**), hypertonic (**b**) and blastocoel fluid (**c**). **d**, Spread of data points comparing the change in *snai2* expression at stage 14. **e**, Schematic of reinflating an embryo to normal blastocoel size after deflation with a saline solution. **f**, Spread of data points comparing changes in *snai2* expression at stage 14. **g**,**i**, Left to right: the schematic of DLMZ graft assay into stage 12 and 10 embryos, respectively and analysed via ISH for *snai2* at stages 18 and 16, respectively. **h**,**j**, Quantification of neural crest competence assay during deflation (**h**) and inflation (**j**) of *Xenopus* embryos. Scale bar, 450 µm (**g**,**i**). Data are mean and s.d. Statistical analysis was performed using two-sided Dunn’s test and unpaired *t*-tests (NS, *P* = 0.2590 (**f**), ***P = 0.0001 (**d**), ****P ≤ 0.0001 (**d**,**f**,**h**,**j**), 95% CI). *n* = 9_control_, 10_hypotonic_, 8_hypertonic_ and 13_blastocoel_ embryos (**d**), *n* = 10_control_, 15_deflation_ and 23_reinflation_ embryos (**f**), *n*_competence_ = 16_control and deflation_ embryos (**h**), *n*_competence_ = 18_control and inflation_ embryos (**j**). Source data

To test the possibility that the osmolarity change resulting from injecting liquid into the cavity influences neural crest induction, we repeated our inflation experiments by injecting hypotonic and hypertonic solutions (Fig. 3a,b), which produced the same inhibition in neural crest induction (Fig. 3d). Furthermore, when injecting the content of a blastocoel cavity taken from another embryo, which corresponds to an equivalent isotonic solution (Fig. 3c), we again observed a reduction in the territory expressing the neural marker *snai2* (Fig. 3d). These experiments suggest that changes in the osmolarity of the blastocoel are not the central factor in controlling neural crest induction. Additionally, it is possible that aspirating blastocoel fluid during deflation causes depletion of an inhibitor of neural crest induction present in the blastocoel. To test this possibility, we repeated our deflation experiments but then reinflated the cavity with a saline solution until the cavity returned to its original volume (Fig. 3e). After blastocoel reinflation, the expression of the neural crest makers was also restored to normal levels (Fig. 3f), ruling out the presence of a neural crest inhibitor in the blastocoel fluid. This experiment resulted in an embryo with a normal-sized blastocoel cavity but with a chemically and osmotically altered constitution. Despite these changes, we observed that neural crest induction was normal, strengthening the notion that osmolarity does not affect neural crest induction, as concluded from the previous experiment (Fig. 3a–d).

**Extended Data Fig. 6:**
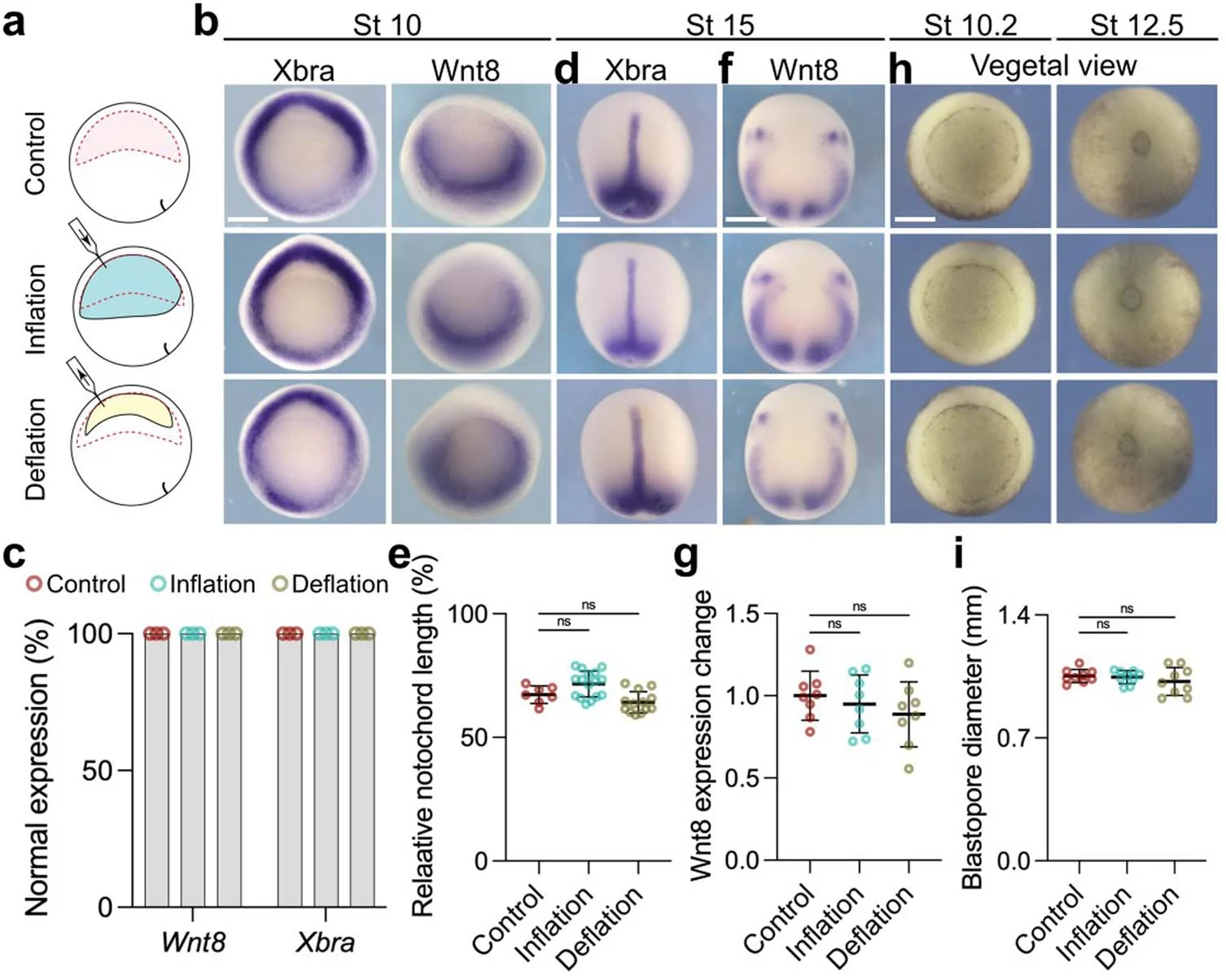
Gastrulation is not affected by changes in hydrostatic pressure. **a**, Schematic of mechanically changing blastocoel volume. **b**. In situ hybridization of embryos at stage 10 analyzing the pan-mesodermal markers *Xbra* and the neural crest inducer gene *Wnt8*. **c**, Percentage of embryos expressing mesodermal markers comparing control to inflated and deflated embryos. **d**, In situ hybridization of embryos at stage 14 analyzing the pan-mesodermal marker *Xbra* and neural crest marker gene *Wnt8*. **e**, Spread of data points indicating the change in notochord length normalized to total length in control (*n* = 7 embryos), inflation (*n* = 16 embryos), and deflation (*n* = 13 embryos). **f**, Effect of inflation and deflation on mesoderm ability to produce *Wnt8*. **g**, Spread of data points comparing change in *Wnt8* expression in control (*n* = 8 embryos), inflation (*n* = 8 embryos), and deflation (*n* = 8 embryos). **h**, Blastopore formation and closure after inflation and deflation compared to control. **i**, Diameter of blastopore closure in control (*n* = 9 embryos), inflation (*n* = 8 embryos), and deflation (*n* = 9 embryos). Scale bar 350 µm (**b**, **d**, **f**, and **h**). Data (**e**, **g**, and **i**) represent mean, error bars are s.d. Statistical analysis was performed using two-sided Dunnett’s test (NS, *P* > 0.05 **e**, **g**, and **i**, CI = 95%). Three independent experiments (**b** and **d**). Source data

**Extended Data Fig. 7:**
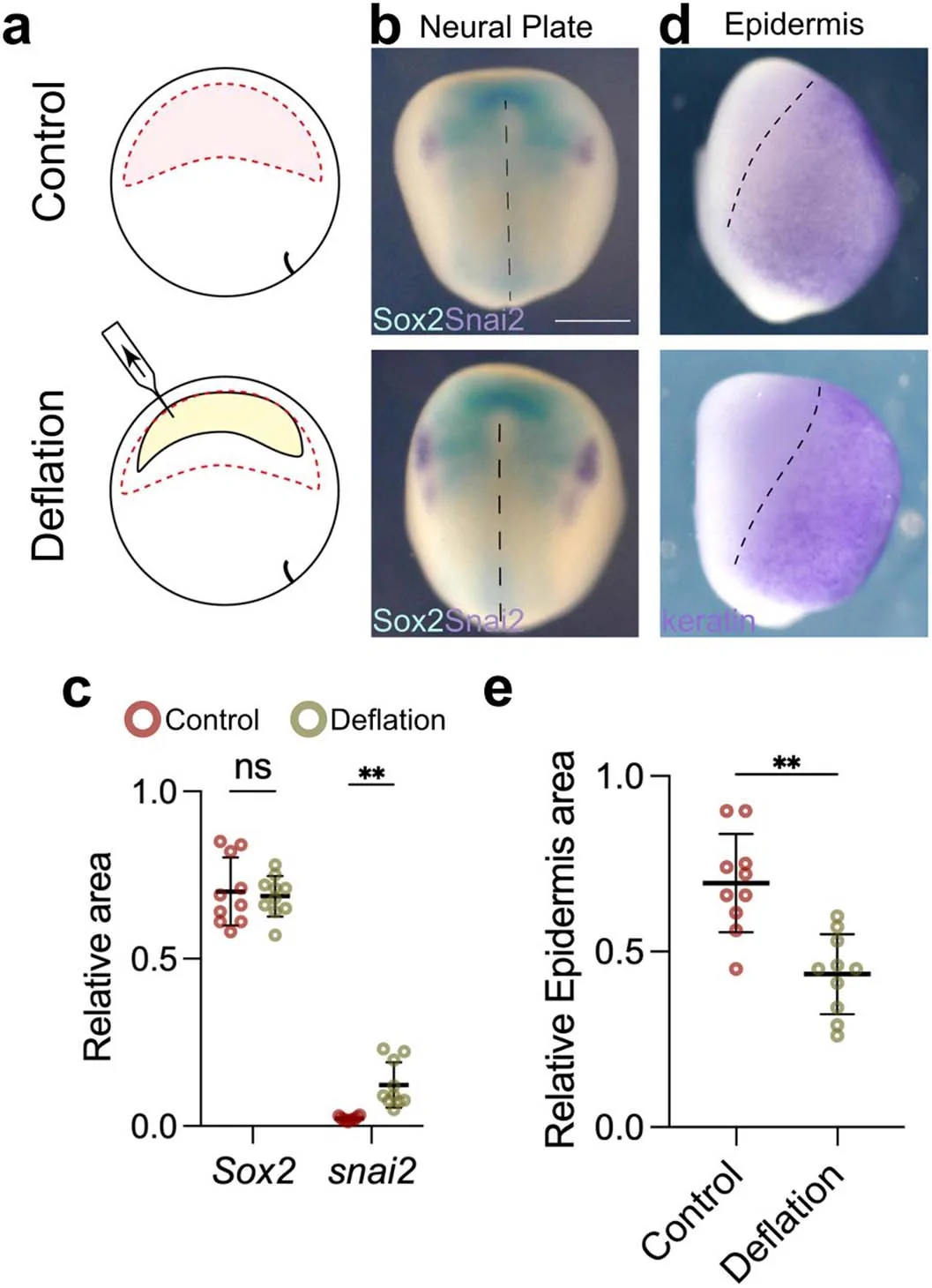
Deflation expands neural crest at the expense of epidermis. **a**, Schematic of deflation assay to reduce blastocoel volume. **b**, Double in situ hybridization of neural plate marker (*Sox2*, cyan) and neural crest marker (*snai2*, purple). **c**, Spread of data points comparing the relative area of the neural plate in control (*n* = 8 embryos) and deflation (*n* = 11 embryos). **d**, In situ hybridization of epidermis marker (*keratin*, purple). **e**, Spread of data points of relative area of epidermis in control (*n* = 10 embryos), and deflation (*n* = 20 embryos). Embryos in (**b**) and (**d**) were analyzed at stage 14. Scale bar 450 µm (**b**). Data (**c, e**) represent mean, error bars are s.d. Statistical analysis was performed using a two-sided unpaired student *t*-test (NS, *P* = 0.0626; **c** and **P = 0.0017; **c** and **e**, CI = 95%). Source data

We next considered that our treatments might affect another tissue and indirectly modify neural crest induction. The mesoderm plays a crucial role in neural crest induction, as it expresses *Wnt8*, a major neural crest inductive signal^13,14,16^. However, we observed no change in the expression of the pan-mesodermal marker *Xbra* nor in the neural crest inducer *Wnt8* at different stages of development after inflation and deflation (Extended Data Fig. 6a–c), indicating that mesoderm was not being affected. Furthermore, no effect was observed in the length of the notochord (Extended Data Fig. 6d,e) or the position or levels of *Wnt8* at stage 15 (Extended Data Fig. 6f,g) nor in the closure of the blastopore (Extended Data Fig. 6h,i), demonstrating that gastrulation had not been disrupted. We also analysed other ectodermal markers and found that the expansion of the neural crest produced by deflation occurs at the expense of the epidermis but not of the neural plate, as shown by the analysis of the neural plate and epidermal markers upon deflation (Extended Data Fig. 7). These observations suggest a specific effect of hydrostatic pressure on neural crest derived from the ectoderm.

**Extended Data Fig. 8:**
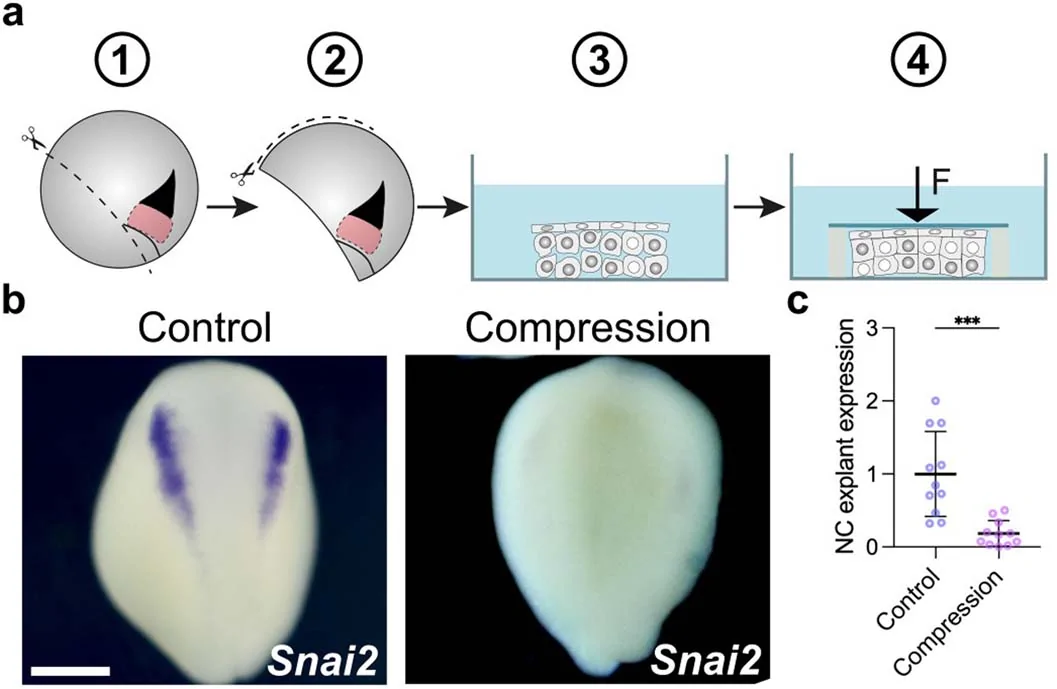
Mechanical pressure inhibits neural crest. Schematic of *in vitro* compression assay of neural crest explants; explants with prospective neural crest were obtained by initial dissection from dorsal blastopore lip to ventral side followed by dissection from animal pole to ventral side. Explants were then compressed under a coverslip, and compressed explants were cultured until stage 18, and the expression of the neural crest marker *snai2* was analyzed. **b**, Representative examples of control (not compressed) and experimental (compressed) explants showing *snai2* expression. **c**, Spread of data points comparing the neural crest expression marker in control (*n* = 11 explants) and compressed (*n* = 11 explants) conditions. Scale bar 400 µm (**b**). Data represent mean, error bars are s.d. Statistical analysis was performed using a two-sided unpaired student *t*-test (***P = 0.001; **c**, CI = 95%). Source data

Finally, as our results suggest that the neural crest can respond to pressure, we directly tested this possibility by applying mechanical pressure to open-faced explants containing the prospective neural crest and the DLMZ. We found that neural crest markers were inhibited in compressed explants when compared with uncompressed control explants (Extended Data Fig. 8). Together, these results indicate that prospective neural crest can respond to pressure (hydrostatic or mechanical) and that an increase in pressure inhibits neural crest induction. This conclusion led us directly to test the hypothesis that neural crest competence is lost due to an increase in blastocoel hydrostatic pressure during normal development. Stage 12 embryos that have lost neural crest competence received a graft of DLMZ, as described in Fig. 1, followed by a decrease in hydrostatic pressure due to deflation (Fig. 3g). While control embryos that received the DLMZ did not express neural crest markers in the site of induction, deflated embryos did (Fig. 3g, h). Furthermore, competent stage 10 embryos showed ventral neural crest induction after grafting DLMZ, whereas the same grafted embryos exhibited no ectopic neural crest induction after blastocoel cavity inflation (Fig. 3i,j). Together, these results demonstrate that simple mechanical manipulation of hydrostatic pressure can modify neural crest competence to DLMZ. Hence, we conclude that neural crest competence is lost due to an increase in the hydrostatic pressure of the blastocoel cavity during gastrulation.

### Hydrostatic pressure regulates the response to Wnt signalling

**Fig. 4:**
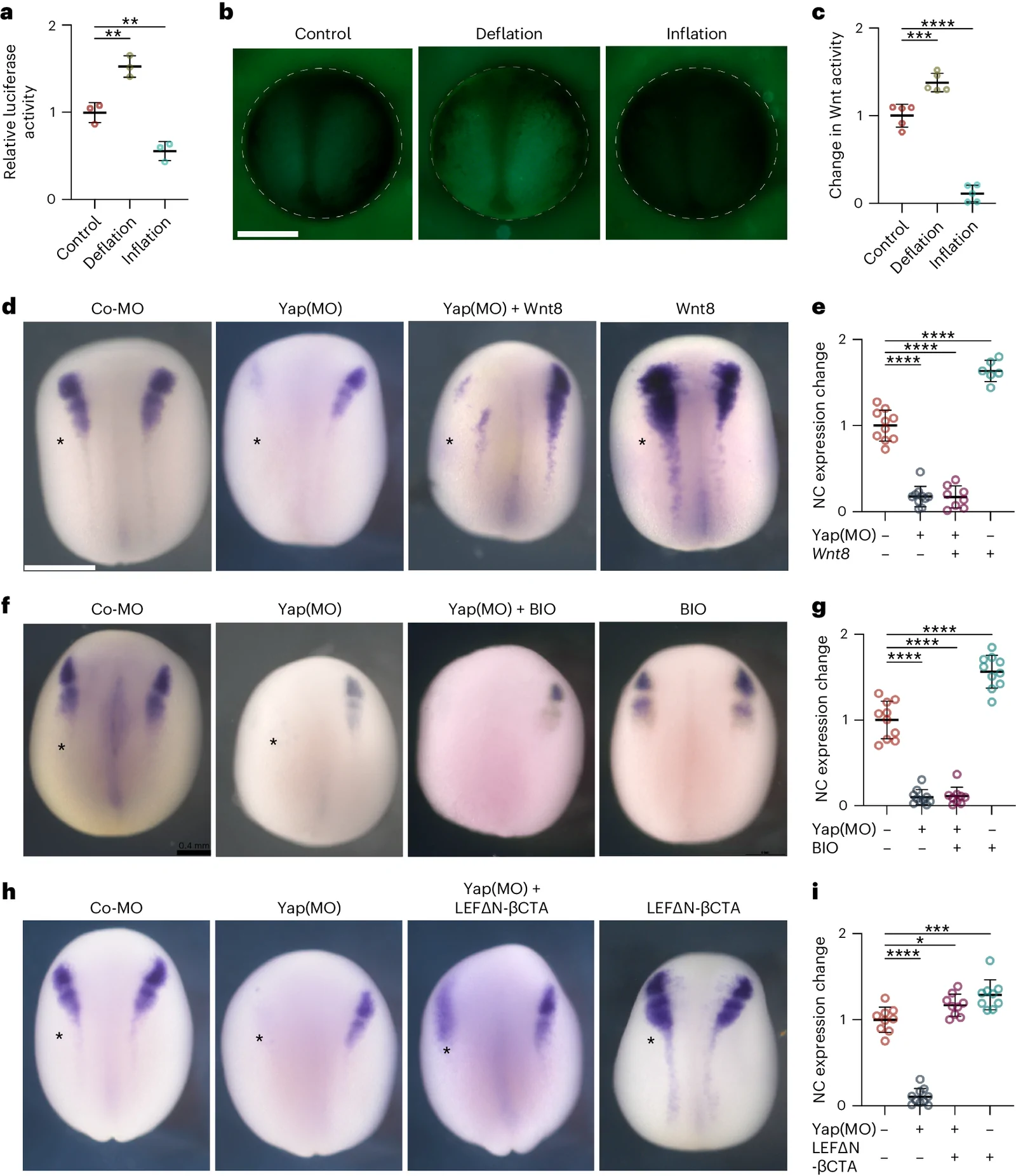
Hydrostatic pressure regulates Yap activity. **a**, Relative luciferase activity of super Top flash after the indicated treatments, normalized to Fop flash. **b**, *Xenopus* transgenic embryos *Tg(pbin7Lef-dGFP)* to detect Wnt activity at stage 12.5 after the indicated treatments. **c**, Quantification of GFP intensity normalized to control embryos. **d**,**f**,**h**, In situ hybridization of *snai2* at stages 16 and 17, after the indicated injections at the eight-cell stage. Asterisks indicate the injected side. Embryos injected with control Yap morpholino (Co-MO), Yap morpholino (Yap(MO)), or Wnt pathway activators. **e**,**g**,**i**, Quantification of *snai2* expression level analysed via ISH during inhibition of Yap, and with *Wnt8* mRNA (**e**), BIO (**g**), or an active form of β-catenin (LEFΔN-βCTA; **i**). Scale bars, 450 µm. Data are mean and s.d. Three independent experiments; each point represents three replicates (**a**). *n* = 5 embryos for each condition (**c**). Statistical analysis was performed using two-sided unpaired Dunnett’s tests (*P = 0.0378 (**i**), **P ≤ 0.0062 (**a**), ***P = 0.0003 (**c**,**i**), ****P ≤ 0.0001 (**c**,**e**,**g**,**i**), 95% CI). *n* = 10_control_, 10_Yap-MO_, 8_Yap-MO + Xwnt8_, 6_Xwnt8_ embryos (**e**). *n* = 10_control_, 10_Yap-MO_, 9_Yap-MO + BIO_, 10_BIO_ embryos (**g**). *n* = 9_control_, 10_Yap-MO_, 9_Yap-MO + β-cat_, 9_β-cat_ embryos (**l**). Source data

**Extended Data Fig. 9:**
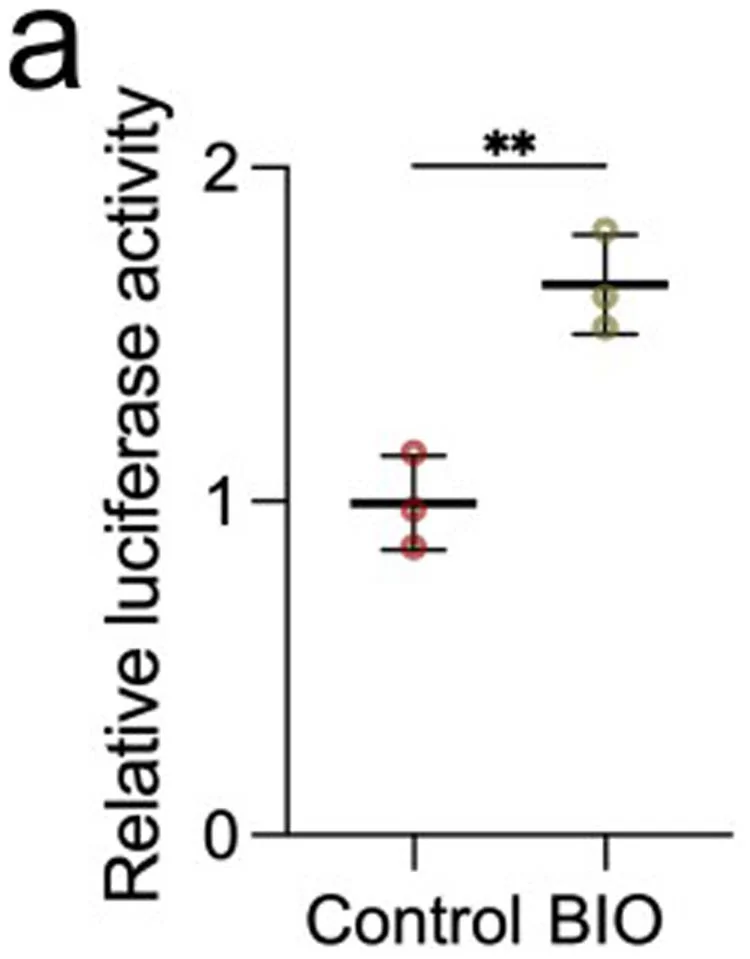
Control for TOP-flash assay. **a**, Data spread indicates relative Wnt activity in control embryos and in embryos treated with BIO. BIO is an inhibitor of GSK3, which should lead to an increase in Wnt activity. Each data point represents three replicates, and each condition represents three independent experiments. Data represent mean, error bars are s.d. Statistical analysis was performed using two-sided student *t*-test (**P = 0.0053, CI = 95%). Source data

Having established that hydrostatic pressure can regulate neural crest competence to DLMZ, we next explored potential mechanisms by which this occurs. Neural crest is induced by a combination of signalling pathways^22^, Wnt signalling being one of the key factors^13,14,16^. Given this, we asked whether hydrostatic pressure could regulate Wnt activity in the responding ectoderm that includes the prospective neural crest cells. To test this idea, we used two approaches to directly measure Wnt activity in the ectoderm in vivo. We expressed in the ectoderm two Wnt sensors in which a promoter containing several Wnt-responsive elements drives the expression of the reporters luciferase^23^ or green fluorescent protein (GFP)^24^. Controls indicate that these are good indicators of Wnt activity (Extended Data Fig. 9a). Both reporters showed that deflation leads to a strong increase in Wnt activity in the neural fold region, whereas inflation inhibits Wnt activity in the embryos (Fig. 4a–c). Together, these experiments show that changes in the blastocoel hydrostatic pressure can modulate Wnt activity in the prospective neural fold area, consistent with the role of blastocoel pressure as a regulator of Wnt response and neural crest competence.

### Yap modulates Wnt during neural crest competence loss

We next investigated the molecular mechanism by which hydrostatic pressure regulates Wnt signalling in the prospective neural crest. Yap has been shown to be a mechanosensory factor that interacts with the Wnt signalling pathway^25–27^, and therefore is a good candidate for modulating Wnt activity during neural crest induction. Consistent with this hypothesis, we observed that morpholino-mediated inhibition of Yap^28^ impairs neural crest formation (Fig. 4d,e). To study possible crosstalk between Yap and Wnt pathways during neural crest formation, we also tested whether activation of Wnt could rescue neural crest induction in Yap-depleted embryos. We found that expression of Xwnt8 (Fig. 4d,e) was not able to rescue neural crest formation in Yap morphant embryos, nor was the GSK3 inhibitor BIO (Fig. 4f,g). This suggests that Yap is unlikely to modulate Wnt signalling at the level of extracellular Wnt, Wnt receptor, or through degradation of β-catenin by GSK3 phosphorylation, but instead must act downstream. All these treatments activating Wnt result in the accumulation of β-catenin in the cytoplasm, necessitating its transfer into the nucleus. To examine the role of nuclear β-catenin in neural crest induction, we introduced a constitutively nuclear variant of β-catenin, termed LEFΔN-βCTA^29^, wherein its transactivation domain is fused to LEF-1, housing a nuclear localization signal. Crucially, the expression of LEFΔN-βCTA successfully restores neural crest induction in Yap-deficient embryos (Fig. 4h,i), indicating that Yap influences the Wnt pathway at the β-catenin level and hinting at Yap potential to facilitate the nuclear migration of β-catenin during neural crest induction.

### Hydrostatic pressure controls Yap activity

**Fig. 5:**
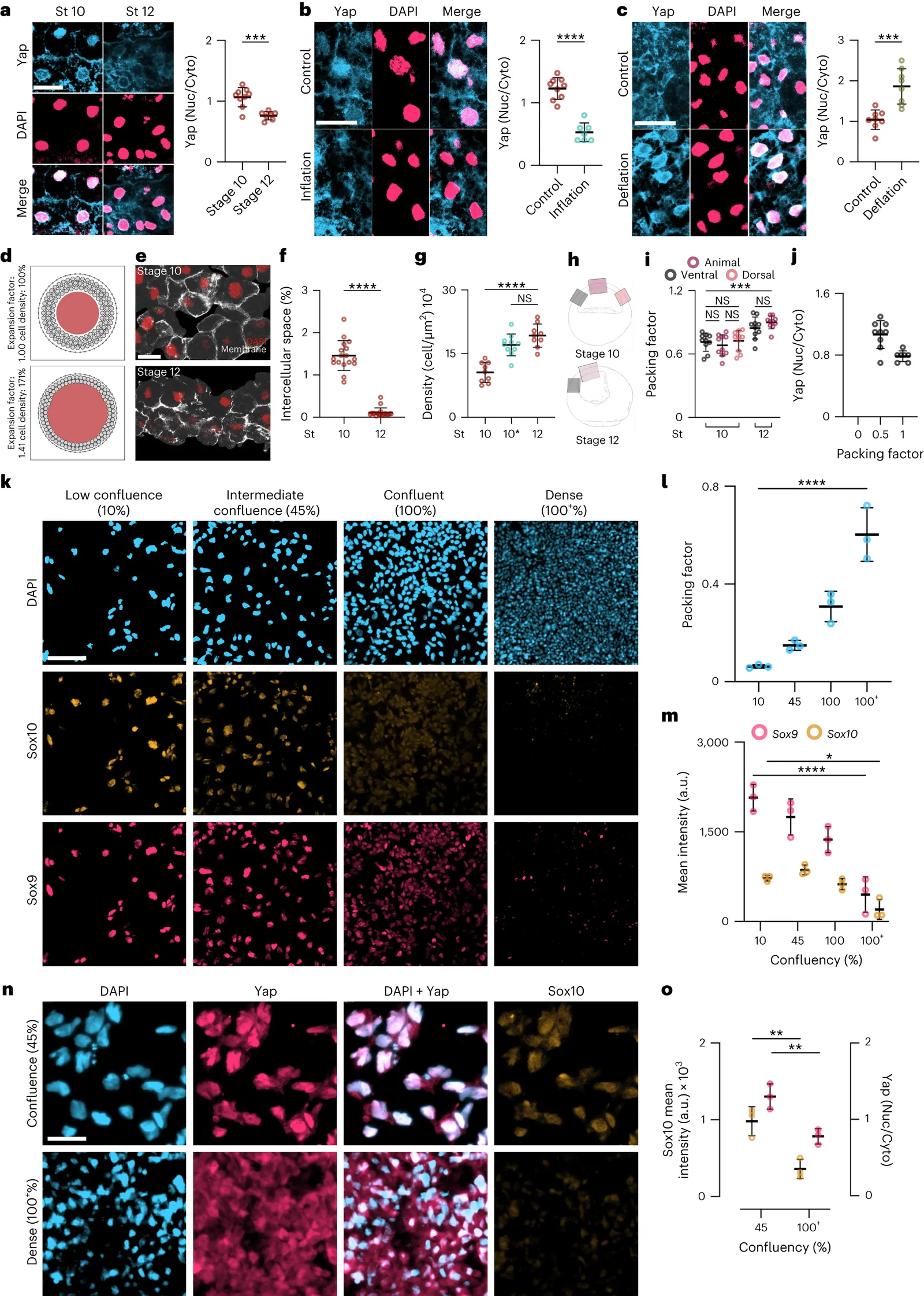
Hydrostatic pressure controls Yap localization. **a**–**c**, Immunofluorescence of ectoderm showing Yap (blue) and DAPI (pink) during gastrulation (**a**) inflation (**b**), and deflation (**c**); graphs show quantification of the Yap nuclear-to-cytoplasmic (Nuc/Cyto) ratio. **d**, Particle-based 2D model illustrating how an expanding cavity (red) can compress surrounding cells (light-grey circles) if they are encapsulated by a stiff shell (the vitelline membrane, dark-grey outer layer). Dark-grey connection lines between cells indicate adhesive interactions. **e**, Immunofluorescence sections of the ectoderm expressing membrane GFP (grey) and stained for DAPI (red). **f**,**g**, Spread of data showing the percentage of intercellular space (**f**) and density (**g**). 10* indicates inflated embryos at stage 10 (cyan) and control embryos (red) at the indicated stages. **h**, Schematic of embryos indicating the regions of interest of ectoderm for analysis of ectoderm packing quantified in **i**. **j**, Spread of data indicating the relationship of ectoderm packing and Yap localization. **k**, Immunofluorescence of different confluences of iNCCs expressing Sox9 (pink) and Sox10 (orange). **l**,**m**, Spread of data showing different cell packing and mean fluorescence intensity at different densities. **n**, Immunofluorescence of Yap (pink) and Sox10 (orange) localization at different confluences of iNCCs. **o**, Spread of data showing mean fluorescence intensity of Sox10 and Yap at different densities. Scale bars, 20 µm (**a**–**c**,**e**), 100 µm (**k**) and 65 µm (**n**). Data are mean and s.d. Statistical analysis was performed using a two-sided two-tailed Student’s *t*-test and Dunnett’s test; (NS, *P* ≥ 0.1408 (**j**,**l**), *P = 0.0168 (**m**), ***P ≤ 0.0010 (**a**–**c,i**) and ****P = 0.0001 (**b**,**f**,**g**,**l**,**m**), 95% CI). Three independent experiments (**i**,**j**,**l**,**m**,**o**). *n* = 9_st10_, 6_st12_ embryos (**a**), *n* = 9_control_, 7_inflation_ embryos (**b**), *n* = 8_control_, 8_deflation_ embryos (**c**), *n*_st10_ and *n*_st12_ = 20 embryos (**f**), and *n*_st10_ = 9, *n*_st10*_ = 10, *n*_st12_ = 9 embryos (**g**). Source data

Having shown that both hydrostatic pressure and Yap modulate Wnt signalling, we next investigated whether Yap is a mechanotransducer that modulates neural crest competence upstream of the response to Wnt signalling. Given that neural crest competence to a DLMZ graft is lost between stages 10 and 12 (N&F) and that nuclear localization of Yap can be used as a proxy for Yap activity^30^, we performed immunofluorescence against Yap and studied its subcellular localization. Competent stage 10 ectoderm showed nuclear localization of Yap, which decreased at stage 12 when competence is lost (Fig. 5a). In addition, increasing hydrostatic pressure by inflating the blastocoel cavity of stage 10 embryos decreased nuclear localization of Yap (Fig. 5b); while decreasing hydrostatic pressure by deflating the blastocoel cavity of stage 12 embryos increased the amount of Yap in the nucleus (Fig. 5c). As increased cell density (cell packing) has been previously shown to drive cytoplasmic localization of Yap in other cell types^31–33^, we asked whether the localization of Yap was dependent on cell packing driven by increased hydrostatic pressure. To test the feasibility of this notion, we performed a computational model that predicted that an increase in the blastocoel volume leads to cells becoming more packed and connected to neighbouring cells (Fig. 5d). We tested this prediction analysing cell packing during development. Non-competent stage 12 embryos exhibited a decrease in the intercellular space and an increase in cell density or packing when compared with competent stage 10 embryos (Fig. 5e–g), regardless of the ectoderm region that was analysed (Fig. 5h,i) This increase in cell density was also observed in embryos in which hydrostatic pressure was increased by inflation (Fig. 5g, stage 10*), indicating that the loss of nuclear Yap correlates with an increase in cell packing (Fig. 5j). To directly test whether cell packing controls nuclear Yap in neural crest cells we induced human iPS cells to become neural crest by Wnt activation^34^, as it is easier to control cell packing by varying the cell density of the culture. Neural crest markers and Yap localization were analysed. We found that increasing cell packing impairs neural crest induction (Fig. 5k–m) and leads to cytoplasmic localization of Yap (Fig. 5n,o), consistent with the idea that in the embryo a rise in hydrostatic pressure increases cell packing, leading to Yap inactivation and loss of neural crest competence.

**Fig. 6:**
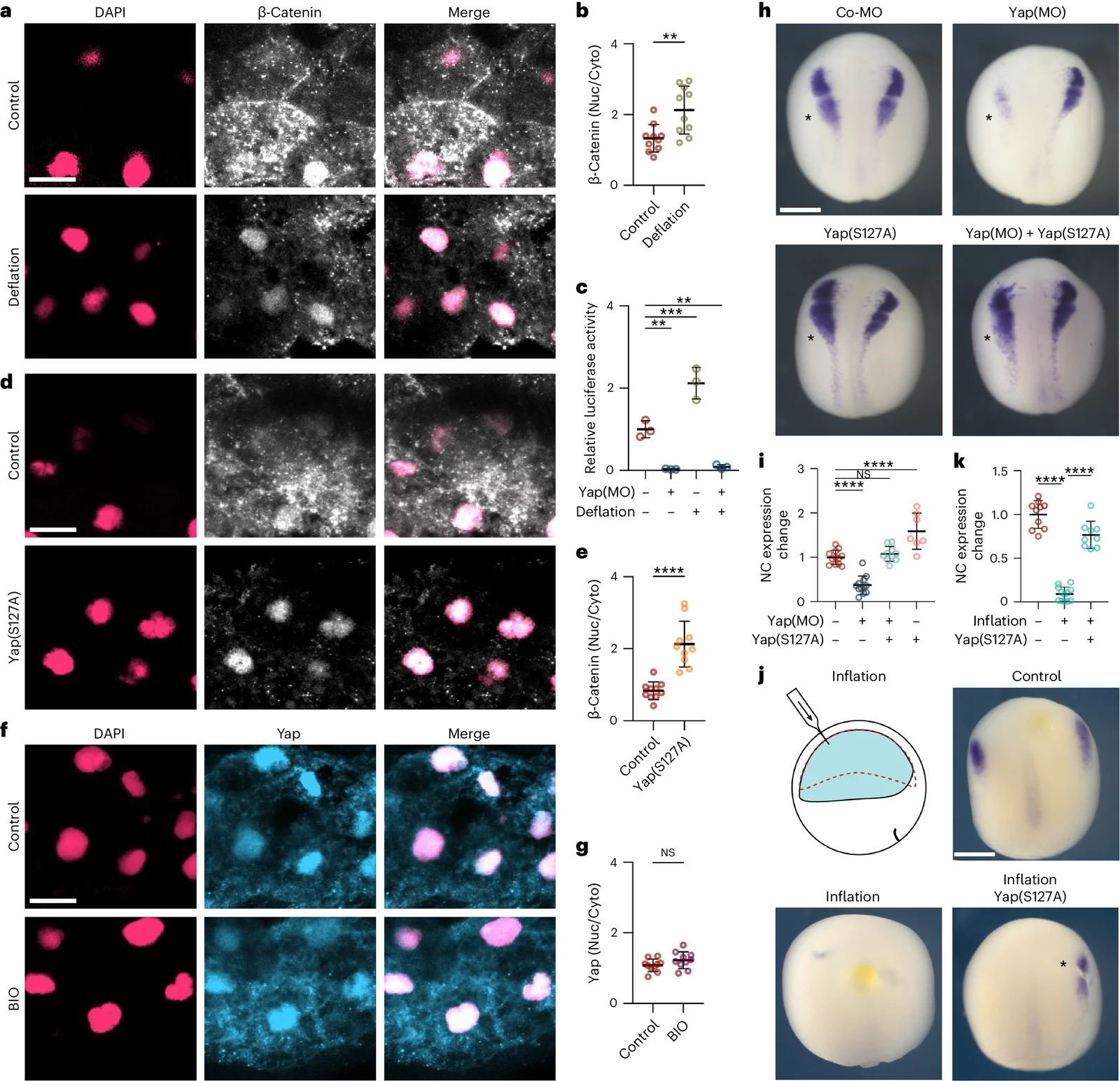
Nuclear Yap mediated by hydrostatic pressure controls Wnt activity. **a**,**d**, Immunofluorescence of β-catenin (pink; DAPI, grey; β-catenin) after deflating or injecting with Yap(S127A) at the 8-cell stage. **b**,**e**, Quantification of the β-catenin nuclear-to-cytoplasmic ratio after treatment of deflation (**b**) and Yap(S127A) (**e**). **c**, Relative luciferase activity at indicated treatments. **f**, Immunofluorescence of Yap after activation of Wnt pathway. **g**, Quantification of Yap nuclear-to-cytoplasmic ratio after treatment. **h**, In situ hybridization of embryos at stage 18 analysing *snai2* after the indicated treatments; asterisks indicate injected side. **i**,**k**, Normalized quantification of *snai2* expression levels after treating *Xenopus* embryos with active form of Yap (Yap(S127A)) either with Yap inhibitor (Yap(MO)) (**i**) or by inflation (**k**). **j**, Schematic of inflation assay and in situ hybridization analysis of *snai2* at stage 14. Scale bars, 15 µm (**a**,**d**,**f**) and 400 µm (**h**,**j**). Data are mean and s.d. Statistical analysis was performed using two-sided unpaired *t*-test and two-sided Dunnett’s tests (NS, *P* ≥ 0.1345 (**i**,**j**), **P ≤ 0.0046 (**b** and **c**), ***P ≤ 0.0006 (**c**), ****P = 0.0001 (**e**,**i**,**k**), 95% CI). *n* = 10 embryos (**b**,**e**,**g**). Three independent experiments; each point represents three replicates (**c**). *n* = 13_control_, 10_Yap-MO_, 12_Yap-MO + Yap(S127A)_, 7_Yap(S127A)_ embryos (**i**). *n* = 10_control_, 12_inflation_, 9_inflation + Yap(S127A)_ embryos (**k**). Source data

To investigate the mechanistic link between Yap activation and Wnt signalling via nuclear translocation of β-catenin, we began by examining the subcellular localization of β-catenin after mechanical deflation of stage 12 embryos. Our findings reveal an increase in nuclear β-catenin post-deflation (Fig. 6a, b), concomitant with the heightened activation of Wnt signalling, as evidenced by luciferase assay results (Fig. 6c). In addition, we observed inhibition of this augmented Wnt signalling in the presence of Yap morpholino (Fig. 6c), strongly suggesting that Yap is indispensable as a mechanotransducer bridging blastocoel hydrostatic pressure to Wnt signalling activation. To further affirm the hierarchical association between Yap and β-catenin, we used the Yap(S127A) mutant—known for its constitutive nuclear localization^30,35,36^—resulting in a demonstrable promotion of nuclear β-catenin (Fig. 6d,e). However, while the GSK3 inhibitor BIO effectively triggered Wnt signalling activation, it did not induce Yap nuclear translocation (Fig. 6f,g), conclusively establishing that Wnt signalling does not precede Yap activity in this context. Collectively, these outcomes demonstrate the essentiality of nuclear Yap in the mechanical regulation of Wnt signalling.

**Extended Data Fig. 10:**
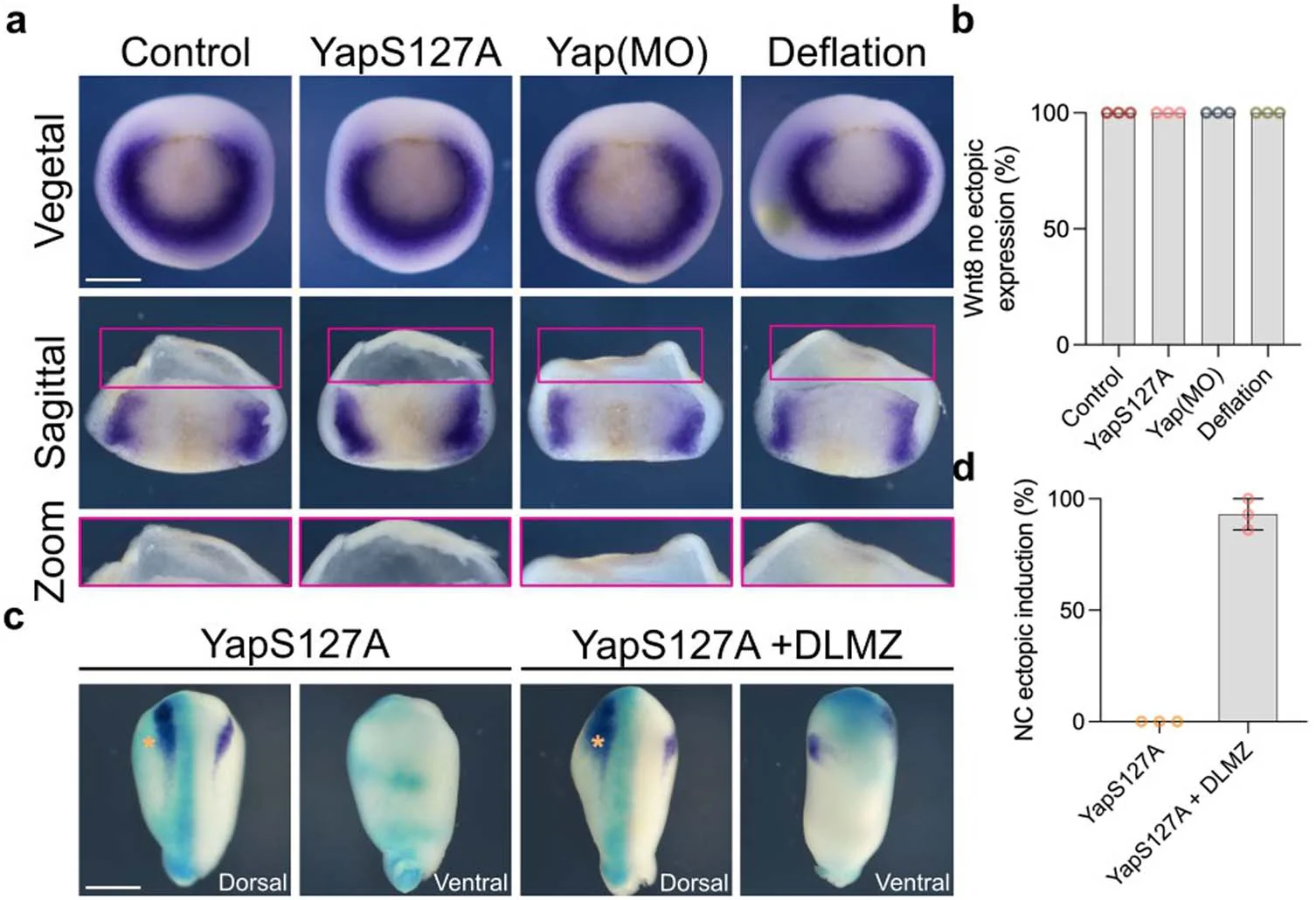
Neural crest ectopic induction mediated by nuclear Yap. **a**, In situ hybridization of mesodermal marker (Wnt8) at different treatments. **b**, Percentage of no observable ectopic Wnt8 expression. **c**, Competence assay analyzed via in situ hybridization. Co-injection on one side of embryos of FLDX (cyan) and active Yap analyzed via *snai2* (purple) at stage 17. **d**, Percentage of ectopic neural crest expression. Scale bar 450 µm (**a** and **c**). Data (**d**) represent mean, error bars are s.d. Three independent experiments. Source data

To directly test the role of nuclear Yap in neural crest induction and competence, we expressed nuclear Yap(S127A) in Yap-morphant embryos, showing that nuclear Yap can rescue inhibition of neural crest induction produced by Yap morpholino, and in addition nuclear Yap leads to neural crest expansion in control embryos (Fig. 6h,i), showing that nuclear Yap is required in the ectodermal cells to induce neural crest. This effect on neural crest induction cannot be explained by modulation of *Wnt8* expression produced by nuclear Yap, as when Yap(S127A) was expressed in the embryo no change in the endogenous level of *Wnt8*, nor ectopic expression was observed (Extended Data Fig. 10a,b). In addition, while DLMZ is able to induce ventral ectopic expression of neural crest markers, nuclear Yap by itself is not able to do so (Extended Data Fig. 10c,d), indicating that nuclear Yap does not work as neural crest inducer, but as a modifier of the proper neural crest inducing signal. Furthermore, we found that inhibition of neural crest by increasing hydrostatic pressure can be rescued by expressing Yap(S127A) (Fig. 6j,k). These results further support the hypothesis that Yap activity can be controlled by pressure.

### Nuclear Yap and β-catenin extends neural crest competence

**Fig. 7:**
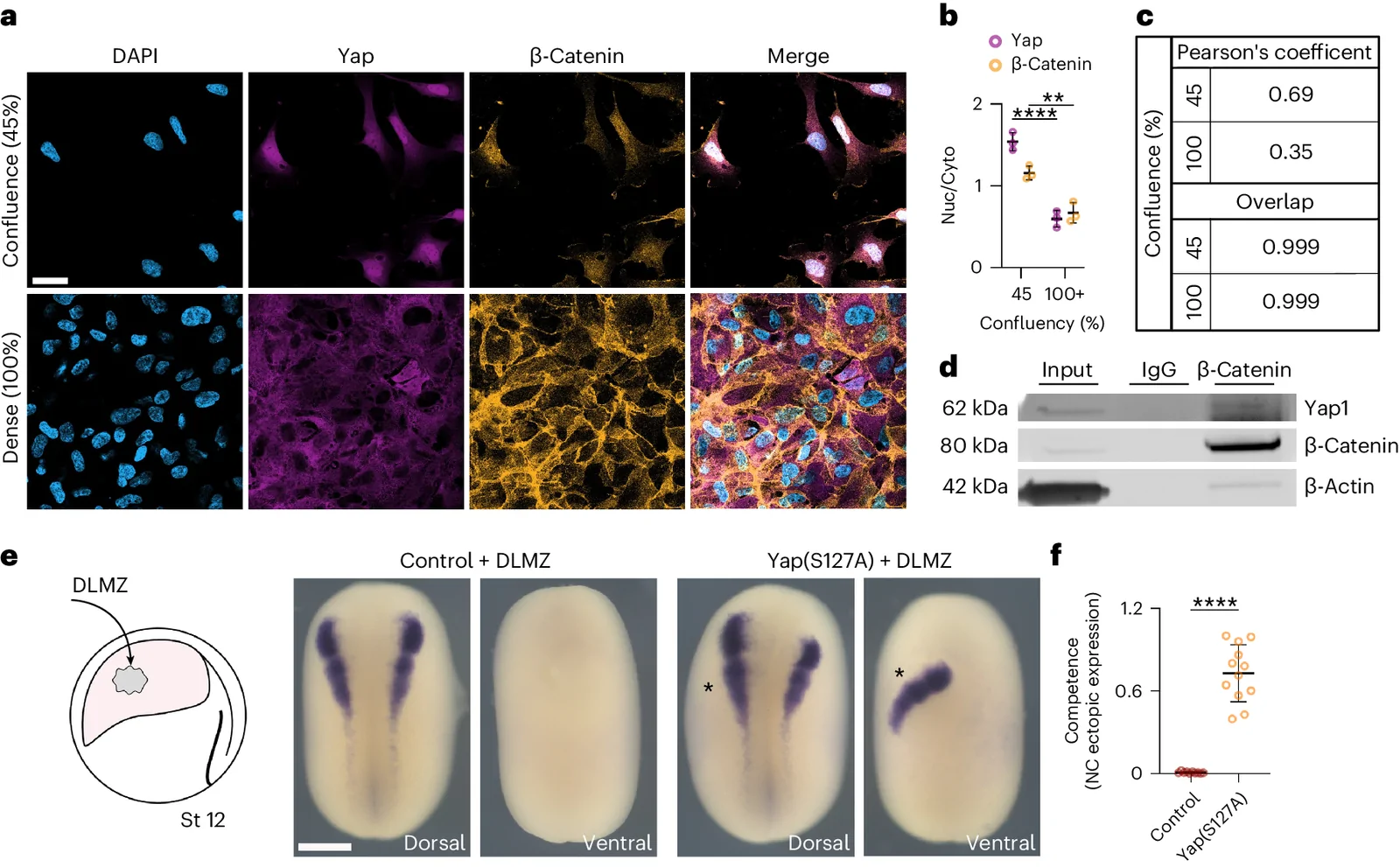
Nuclear Yap and β-catenin extends neural crest competence. **a**, Immunofluorescence of iNCCs at different confluency stained for β-catenin and Yap. **b**, Quantification of Yap nuclear-to-cytoplasmic ratio. **c**, Pearson’s coefficient and overlap of β-catenin and Yap at different confluencies. **d**, Co-immunoprecipitation analysis of β-catenin, no specific binding IgG. **e**, Schematic of neural crest competence assay and in situ hybridization analysis of *snai2* at stage 18. **f**, Quantification of neural crest competence normalized to control. Scale bars, 20 µm (**a**) and 400 µm (**e**). Data are mean and s.d. Statistical analysis was performed using ordinary two-way ANOVA and two-sided unpaired *t*-test (**P ≤ 0.0058 (**b**), ****P = 0.0001 (**b**,**f**), 95% CI). Three independent experiments (**b**,**c**). *n* = 11_control_, 12_Yap(S127A)_ embryos (**f**). Three independent experiments (**d**). An unprocessed blot is available as Source data. Source data

We have shown that nuclear Yap is upstream of Wnt signalling during neural crest competence and that lack of Yap protein can be rescued by nuclear β-catenin, but not by its cytoplasmic accumulation. Together, these observations suggest that nuclear Yap enables the activation of Wnt signalling by controlling β-catenin nuclear transportation. This idea is supported by the observation that mechanical activation of Yap (by low cell density) in Wnt-induced iPS cells leads to nuclear co-localization of Yap and β-catenin (Fig. 7a–c) and that Yap protein co-immunoprecipitates with β-catenin showing interaction between these two proteins (Fig. 7d). Finally, to test whether neural crest competence to DLMZ requires nuclear Yap, we performed the DLMZ grafts in non-competent stage 12 embryos previously injected with Yap(S127A). Our results showed that neural crest competence is restored at stage 12 by nuclear Yap (Fig. 7e,f). Together, these results place Yap as a mechanotransduction factor downstream of hydrostatic pressure and show that its nuclear presence confers neural crest competence by promoting nuclear translocation of β-catenin.

## Discussion

The role of mechanical cues, let alone hydrostatic pressure, on embryonic competence has never been investigated before. We show here that the loss of neural crest competence, the capacity of a tissue to respond to inductive signals^4^, results from an increase in hydrostatic pressure in *Xenopus* and mouse embryos and in human neuruloids. In *Xenopus*, this increased hydrostatic pressure in the blastocoel cavity leads to cytoplasmic retention of Yap, which regulates Wnt activity in the responding ectoderm, an essential signalling pathway for neural crest induction^13,14,16^. Embryonic competence is one of the key features that sets embryonic induction apart from cell differentiation. Embryonic induction and cell differentiation have been extensively examined, and various molecules and cell signalling pathways have been identified that regulate these processes. In addition, environmental factors (mechanical inputs) have been shown to contribute to cell differentiation^1,2,6,37^. However, since the proposal of the embryonic competence concept over 100 years ago, investigations have heavily focused only on the role of biochemical signals^7–9^ without finding a mechanism that controls embryonic competence. Thus, we leveraged our molecular understanding of neural crest induction^13,16^ to investigate a possible role of mechanical cues as a mechanism that controls the early embryonic competence of these cells.

The role of hydrostatic pressure in development and cell differentiation has been a topic of growing interest in the past few years, as it has been shown to play an essential role in lumen formation in zebrafish gut and ear, in mammalian lungs, mice blastocyst, in *Drosophila* oogenesis, as well as many others^38^. The increase in *Xenopus* blastocoel volume depends on the activity of the Na^+^,K^+^-ATPase^21^, and it starts at the early segmentation stages and continues gradually until gastrulation^18^. While in many systems a change to the osmotic pressure is the mechanism that leads to cell differentiation, our experiments show that neural crest induction can be affected by modifying hydrostatic pressure regardless of the osmolarity, suggesting that hydrostatic pressure is the main factor in controlling neural crest competence. Furthermore, finding that an increase in mechanical pressure inhibits neural crest induction further supports the notion that the cells sense changes in pressure rather than osmolarity.

Our model prediction and observations are consistent with previous evidence that has shown a higher cell density can lead to cytoplasmic Yap retention in cell cultures, while lower confluency generates cells with nuclear Yap^31–33,39–43^. Thus, we propose that the highly loose ectodermal cells at the competent stages become more packed at the non-competent stages by a rise in hydrostatic pressure leading to an increase in cell density, producing Yap cytoplasmic retention. We observed that, during the increase in blastocoel hydrostatic pressure, there is a surge in cell density, which is consistent with this idea. This increase in cell density by higher hydrostatic pressure from the inner cavity is possible because the embryo is constrained by the vitelline membrane, which does not allow an increase in embryo volume during blastocoel expansion. The observation that *Xenopus* competence is dependent on cell packing controlling Yap, suggests the generality of this mechanism across species, as we observed similar effects of hydrostatic pressure in mouse embryos and human-derived neuruloids leading to the inhibition of neural crest induction. Indeed, a link between Yap activation and hydrostatic pressure has been recently shown in cells cultured in vitro^44^. However, further studies are necessary to fully establish the source of hydrostatic pressure in embryos with a topology different from amphibians. Although our study indicates hydrostatic pressure as the main regulator of Yap activity in our system, we cannot rule out that other mechanical cues^30,31,33,39,40^ could also regulate Yap during embryonic competence, especially considering the diverse topologies of different embryos during neural crest induction^30,31,33,39,40^.

Yap is a well-established mechanosensor downstream of the Hippo pathway and its transcriptional activity and cellular localization are well-accepted as a readout of mechanical signals^42,45,46^. Moreover, the mutual regulation of Yap and Wnt signals has been previously described in other systems, but often with contradictory outcomes^25,26,45,47–50^. For instance, two independent groups demonstrated that Wnt-mediated intestinal tumorigenesis is abolished by deletion of Yap^47,49^, whereas other groups noted that Yap attenuation has a negligible effect on intestinal tumorigenesis^25,26^. Furthermore, in Wnt-driven cellular response, some studies found an increase in Yap target genes^49,51^, whereas some have shown that Yap inhibits the Wnt/β-catenin pathway by promoting the activation of the β-catenin destruction complex^25^. Mechanistically, it has been shown that Hippo/Yap and Wnt/β-catenin interact at different levels; Yap in the cytoplasm interacts with β-catenin, Disheveled and AXIN1 (refs. ^52–54^), and in the nucleus it interacts with β-catenin to promote target genes^55,56^.

Our study findings propose that Yap serves to activate Wnt signalling through the facilitation of nuclear translocation of β-catenin, mirroring observations in various systems^54,57–59^. We demonstrate that, in Yap-depleted embryos, nuclear β-catenin, not its cytoplasmic counterpart, effectively restores neural crest induction. While β-catenin lacks explicit nuclear localization signals, recent evidence points towards its nuclear import being reliant on interactions with Kap-β2 (also known as transportin-1)^60^. Moreover, the overexpression of TCF/LEF-1 prompts β-catenin nuclear accumulation, suggesting TCF/LEF-1 involvement in its nuclear import^61^. Notably, only a fusion construct combining the transactivation domain of β-catenin with the nuclear localization of LEF-1 (LEFΔN-βCTA) effectively reinstates neural crest in Yap-deficient embryos, indicating that the main function of Yap in this system is promoting β-catenin nuclear localization. Additionally, mechanically activated Yap co-localizes with β-catenin in the nucleus, exhibiting interaction; however, the nature of this interaction, whether direct or mediated by other proteins, necessitates further investigation. Thus, the observed increase in hydrostatic pressure during development impedes Yap nuclear presence, consequently hindering the requisite nuclear translocation of β-catenin for neural crest induction, even in the presence of the Wnt inductor. This regulatory mechanism governing the response to inductive signals embodies the hallmark of embryonic competence.

Several studies have investigated Yap function in the development of neural crest cells from its induction, migration and differentiation^62,63^. However, only a few studies aimed to examine the role of Yap in early development^28,64^. Indeed, previous studies suggested Yap has a role in neural plate border where a loss of function led to the inhibition of *Pax3* (a marker for neural plate border and neural crest progenitor) and that the gain of function expanded the *Pax3* domain but simultaneously inhibited neural crest induction^28,64^. The authors suggested that Yap holds the neural plate border in the progenitor state. Unfortunately, these studies are inconclusive, as in addition to modifying neural crest, they also reported an effect on mesoderm, making it impossible to determine whether the effect on neural crest induction is direct or mediated by the mesoderm^28^. Hence, while the role of Yap in neural crest development has been previously acknowledged, this study introduces an original perspective by delving into mechanobiology and unveiling the upstream cues that regulate, via Yap, embryonic competence towards neural crest fate. Notably, it sheds light on the responsiveness to established neural crest-inducing growth factors such as Wnt by demonstrating that Yap activity endows otherwise non-competent ectoderm with the capability to respond to inductive signals. More broadly, we anticipate a general mechanism wherein diverse mechanical stimuli may regulate Yap, influencing its interplay with various signalling pathways and thereby modulating competence across diverse tissues, organs and even species.

## Methods

The research performed in this study complies with all relevant ethical regulations approved by the UK Home Office, and the ethical council from University College London.

### *Xenopus laevis* and mice work

*Xenopus* embryos were obtained as previously described^65^. In brief, superovulation of a mature female was induced by injecting subcutaneously 100 IU of pregnant mare serum gonadotrophin (Intervet) into the dorsal lymph sac. A second injection (after 72 h) with 250–350 IU of human chorionic gonadotrophin (Intervet). Eggs were fertilized with a mixture of sperm solution. Male testis was obtained from European Xenopus Resource Center (EXRC). *Xenopus* embryos were staged in accordance with the previous work^15^. Embryos were dejellied with 2%(w/v) l-cysteine (pH 8.0) and maintained throughout development in 0.1 Marc’s modified Ringer’s medium or in 3/8 Normal Amphibian Media. Animal licences were approved by the Animal Welfare and Ethical Review Board at University College London and issued by the UK Home Office. Mouse work was done at the facility of the biological service unit at University College London, all work was done under guidelines of personal and establishment licence. CD1 pregnant female mice were obtained and killed on day 8.

### Neuruloids and hiNCCs and immunostaining

Neuruloid differentiation was adapted from a previously published method by the substitution of HUESM by E6 medium (Essential 6, ThermoFisher). hiPS cells NIBSC8 obtained from the National Institute for Biological Standards and Control (NIBSC-UK) were maintained in E8 medium (Essential 8, ThermoFisher) on Matrigel (Corning)-coated dishes. For neuruloid differentiation, 150,000 cells ml^−1^ hiPS cells in E8 plus 10 μM Y27632 (Sigma) were plated on coverslips with 500 μm diameter circular patterns. Patterns on coverslips were created by covering the Matrigel-coated coverslip with parafilm with 500 μm punch holes and plating cells on top. After 24 h, parafilm was removed and coverslips were washed with PBS and medium changed to E6 with 10 μM SB431542 (Sigma) and 0.2 μM LDN 193189 (Sigma), in which cells were maintained for 3 days. On day 3, medium was changed to E6 with 10 μM SB431542 and 50 ng ml^−1^ BMP4 (Prepotech). Medium was changed on day 5 using the same medium, and neuruloids were cultured until day 7. For hydrostatic pressure experiments in neuruloids, 15-ml conical tubes (Corning) had the extremities cut and fused using a Bunsen burner to reach 30 cm high. Coverslips with neuruloids on day 5 were transferred to a 15-ml conical tube cap, which was attached to the fused tube and filled up with medium until the 30-cm mark. Neuruloids were recovered on day 7, washed with PBS and fixed in 4% paraformaldehyde. Neuruloids were permeabilized with PBS 0.1% Triton-X and blocked with 5% bovine serum albumin (BSA), then incubated overnight at 4 °C with antibodies for Sox10 (1:50, Developmental Studies Hybridoma Bank (DSHB)) in blocking solution. Secondary antibody incubation was performed for 1 h at room temperature using AlexaFluor 488. Images were taken using a spectral (SP) confocal microscope (Leica) and analysed using FIJI.

Human induced neural crest cells (hiNCCs) were generated by BMP inhibition and Wnt activation in iPS cells as previously described^34^. In brief, iPS cells NIBSC8 maintained in E8 medium were plated on Matrigel-coated wells using hiNCC induction medium for 5 days. Induction medium of hiNCCs was constituted Dulbecco’s modified Eagle medium/F12 GlutaMax (ThermoFisher Scientific), 0.5% BSA (Sigma), 3 μM CHIR99021 (Sigma) and 2% B27 supplement (Thermo Fisher Scientific). Different iPS cell confluences were generated by plating different concentration of iPS cells (5,000, 20,000, 40,000 and 160,000 cells cm^−2^). After 5 days, cells were fixed with 4% paraformaldehyde, permeabilized with PBS 0.1% Triton-X and blocked with 5% BSA. Cells were then incubated overnight at 4 °C with antibodies for Sox9 (1:500, Sigma), Sox10 (1:50, DSHB) and YAP1 (1:200, Proteintech) or anti-β-catenin (BD Transduction Laboratories, 610153) in blocking solution. Secondary antibody incubation was performed for 1 h at room temperature using AlexaFluor 488, AlexaFluor 594 (ThermoFisher) and 4′,6-diamidino-2-phenylindole (DAPI). Cells were imaged with EVOS 7000 and images analysed with FIJI ImageJ.

### Graft and in vitro neural crest experiments

Dorsolateral marginal zone (DLMZ) tissue was dissected from stage 10 (N&F) donor embryo and grafted into the cavity of the host embryos at different gastrulation stages as previously described^11^. The grafted embryos were incubated up to mid-late neurula stages, thereafter fixed, and ectopic induction was analysed by in situ hybridization. Grafted embryos with an ectopic induction that extends to the endogenous neural crest induction, leading to dorsal–lateral ectopic induction, were removed from the analysis, as shown (Extended Data Fig. 1d–f). For explant compression, explants containing the prospective neural crest were obtained by first cutting the ventral tissue (from the dorsal blastopore lip to the animal-ventral side). A second cut was made from the animal pole to the ventral side to open the explants. Then explants were compressed under a coverslip using vacuum grease (Dow Corning, 0315) to control the compression level.

### Mechanical assays

Inflation and deflation were performed by using a microinjector (MicroData Instrument). Microneedles were prepared (pulled, Narishige PC-10, and calibrated via microforge, Narishige, MF2) with 10–20 µm tip to inflate embryos by injecting 100–250 nl of medium inside the blastocoel cavity and, for deflation, a microneedle with 20-µm tip to aspirate 100–175 nl of blastocoel liquid from the blastocoel cavity. Medium with different osmolarity was used for the inflation: hypotonic (0.1 Marc’s modified Ringer’s medium, of 222 mOsm), hypertonic (3% FICOL, Sigma-Aldrich, 900 mOsm), isotonic (fluid was taken from the blastocoel cavity from sibling embryos). Mouse and neuruloid hydrostatic pressure assays were done by placing the mice embryo/neuruloids at the bottom of a graduated cylinder with a median height (*h*) of 30 cm. Mouse embryos were placed in 100% rat serum, and neuruloids were cultured as described above.

### *Xenopus* microinjections and treatments

Microneedles for microinjections were pulled (Narishige, PC-10) and calibrated under a dissecting microscope (LeicaMZ6). Microinjections were performed in one blastomere of eight-cell stage embryos using a PM1000 microinjector (MicroData Instrument) as described previously^28^. Seventy nanograms of a previously characterized Yap morpholino^6^ obtained from GeneTools was injected. The following messenger RNA was injected as described: 2 ng of Yap(S127A), 8 pg of Xwnt8 (ref. ^66^) and 200 pg of β-catenin-GR^29,67^. For the Yap(S127A) construct (Addgene plasmid 17790), the original insert was subcloned into Spe1 and Xba1 sites in pBluescript SK(+) vector, followed by in vitro transcription of this construct and used in this study (mMessage mMachine kit, Thermo Fisher Scientific, AM1340 for SP6 and AM1334 for T7).

Embryos were incubated with 70 mM of the Na^+^,K^+^-ATPase inhibitor ouabain (Sigma-Aldrich, O3125) as previously described^21^ or 25 µM of the GSK3 inhibitor BIO (Sigma-Aldrich, B1686) as described^68^. To induce activated β-catenin-GR, stage 10 embryos were incubated with 4 mg ml^−1^ dexamethasone (Sigma-Aldrich) until stage 17, as previously described^29^.

### In situ hybridization

Whole-mount colorimetric in situ hybridization was performed in accordance with previous protocol^69^. Riboprobe system (Promega P1420) was used to generate digoxigenin antisense probes for *snai2* (ref. ^10^), *foxd3* (ref. ^17^), *keratin*^70^, *Xbra*^71^ and *Wnt8* (ref. ^72^), and a fluorescein antisense probe was generated for double colorimetric in situ hybridization for *Sox2* (ref. ^73^). Embryos were fixed in MEMFA (0.1 M MOPS pH 7.4, 2 mM EGTA, 1 mM MgSO_4_ and 4% paraformaldehyde) dehydrated and bleached, then incubated with the probe (two probes for the double in situ hybridization). Next, embryos were blocked in a 2% blocking reagent, incubated with anti-digoxigenin-AP antibody (Roche) 1:3,000 and revealed with nitro blue tetrazolium (NBT)/5-bromo-4-chloro-3-indolyl-phosphate (BCIP) in alkaline phosphatase (AP) buffer. For double colorimetric staining, embryos were washed, blocked and incubated with anti-fluorescein-AP antibody (Roche) 1:3,000 and revealed first with BCIP in AP buffer. Embryos were then fixed in 3.7% formaldehyde and imaged with Nikon SMZ800N. Then, images were analysed using ImageJ, where a region of interest was selected, and integrated density was measured. Final values were subtracted from the background and then normalized to control.

### Measurement of Wnt activity

Wnt activity was measured by Luciferase assay as previously described^23^. In brief, *Xenopus* embryos were injected into the animal blastomeres at the eight-cell stage, which are fated to become ectoderm. Embryos were co-injected with 100 pg of super Top-flash M50 and Fop-flash M51 DNA (Addgene plasmids 12456 and 12457, respectively). Then embryos were homogenized in triplicate, and samples were lysed using the Luciferase assay system (Promega, E4030). Top-flash activity was measured (Luminometer, Tuaner BioSytem) and normalized to Fop-flash. In addition, a *Xenopus laevis* transgenic line was used to analyse Wnt activity spatially. The transgenic *Xla.Tg(WntREs:deGFP)*^*1Vlemx*^ was used in accordance with previous work^24^. In brief, transgenic testes were obtained and used for in vitro fertilization. Then embryos were incubated to stage 10, where embryos were either inflated or deflated. Then embryos were incubated till stage 12 to ensure signal turnover. Images were then taken in a fluorescent microscope (Nikon SMZ25), and a selection of region of interest in ImageJ measured integrated density; integrated intensity values were normalized to the control values.

### High-resolution micro-CT scan

*Xenopus* embryos were fixed with 4F1G fixative (1% glutaraldehyde and 3.7% formaldehyde in 0.2 M phosphate buffer) overnight and stained with 10% iodine potassium iodide overnight as previously described^74^. Embryos were scanned (XT H 225, Nikon) at 60–80 kV, 8 W and an exposure time of 0.5 s with a 0.250 mm copper filter. Images were reconstructed (inspect-X, Nikon) at a cubic voxel size of 12 µm after 2 × 2 binning. Analysis was performed using gmsh (GNU General Public License, version 4.8.4), an automatic three-dimensional finite element mesh generator for volume analysis. For images, ImageJ was used to mask and reconstruct the three-dimensional volume using the volume viewer plugin.

### RT–qPCR

RNA was extracted from a pool of embryos (20 per sample) using the RNeasy Micro Kit (Qiagen). Complementary DNA synthesis from RNA was performed using VILO cDNA Synthesis Kit (Invitrogen). Reverse transcription quantitative PCR (RT–qPCR) was performed on an Eco Real-Time PCR System (Illumina) using the primers *snai2* (ref. ^75^); Fw: CATGGGAATAAGTGCAACCA, Rev: AGGCACGTGAAGGGTAGAGA and *foxd3* (ref. ^76^); Fw: TCTCTGGGGCAATCACACTC, Rev: GTACATTTGTTGATAAAGGG and the Power SYBR Green PCR Master Mix (Invitrogen). The reaction mixture consisted of Power SYBR Green PCR Master Mix, 250 nM primers, and 4 ml cDNA in a total volume of 20 ml. The PCR conditions were as follows: 95 °C (10 min, 40 cycles) at 95 °C (10 s) and 60 °C (30 s). *odc1* (ref. ^75^) was used for normalization.

### Hydrostatic pressure measurement

To measure the hydrostatic pressure of the blastocoel cavity, a 900 A micropressure system (World Precision Instruments, SYS-900A), which has a resolution of 13 Pa, was used in accordance with previous work^77^. In brief, 0.5–1 μm microneedles (World Precision Instruments, TIP05TW1F, TIP1TW1) were filled with 1 M KCl solution and attached to the microelectrode holder (World Precision Instruments, MEH6SF) and connected to 900 A system. The microelectrode was calibrated using a calibration chamber (World Precision Instruments, CAL900A). The microelectrode was positioned onto a micromanipulator and the reference electrode was mounted in a fixed position inside the media under LeicaMZ6 or a Nikon SMZ645 dissecting microscope. Embryos were fixed in position using plasticine to ensure embryos were stable during pressure measurement. The microelectrode was inserted into the blastocyst at a depth above the ectoderm thickness of 100 µm, around 300 µm (precisely measured using a micromanipulator) and then maintained in place for ~10 s. The pressure was calculated as the mean pressure of 10 s. Data points with decreased pressure within the 10 s of probe insertion failed to stabilize and were discarded due to repature of the ectoderm.

### Immunostaining and cryosection

Embryos were fixed in 4% paraformaldehyde, treated with 0.03% Triton (100-X, Sigma-Aldrich), blocked in 20% normal goat serum (NGS), and incubated with Yap1 antibody 1:200 (Proteintech, 13584-1-AP). A secondary antibody was added AlexaFluro488 or AlexaFluro555 1:360 (Thermo Fisher Scientific) and DAPI 1:1,000 (Sigma-Aldrich, D9542). Then embryos were fixed with 1% paraformaldehyde and then dehydrated with 30% sucrose/PBS. Then, embryos were embedded in with 30% optimal cutting temperature (OCT) and sectioned using a cryostat OTF5000 (Bright Instrument) and imaged using a confocal microscope (TCS SP8; Leica Microsystems). The intensity of Yap staining was measured by ImageJ, and the nuclear-to-cytoplasmic ratio was calculated using a mask based on DAPI staining to identify the nucleus. Then the integrated density of the nucleus was obtained and subtracted from the total to obtain the cytoplasmic value. Cell packing of the ectoderm was measured as previously described^78^: ${\rm{Packing}}\; {\rm{index}}=\frac{{n}_{{{\mathrm{cells}}}}\times {\bar{X}}_{{{\mathrm{cell}}\; {\mathrm{area}}}}}{{{\mathrm{Total}}\; {\mathrm{area}}}}.$

### Co-immunoprecipitation

Pull-down of B-catenin co-interacting proteins was performed followed by sodium dodecyl sulfate polyacrylamide gel electrophoresis and western-blot visualization of Yap. In brief, 5 × 10^6^ hNCCs were lysed in 900 μl of ice-cold lysis/wash buffer (PBS 1% Triton-X, Halt Protease and Phosphatase Inhibitor, ThermoFisher), incubated on ice for 10 min and centrifuged for 10 min at 16,000*g* 4 °C. The supernatant was then incubated for 4 h at 4 °C with Dynabeads Protein G (ThermoFisher) prepared overnight with 50 μg of either B-catenin (BD Biosciences) or mouse IgG (ThermoFisher) antibodies. Cell lysates with Dynabeads were then placed on a magnetic stand and washed three times with ice-cold lysis/wash buffer and proteins were eluted in lithium dodecyl sulfate (LDS) buffer with 100 mM dithiothreitol at 70 °C for 10 min. Cell lysate (input), β-catenin and IgG co-immunoprecipitated proteins were run on a 12% NuPAGE Bis-Tris Gel (ThermoFisher) at 200 V for 60 min and transferred to nitrocellulose membranes using iBlot 2 Transfer System (ThermoFisher). Membranes were blocked for 1 h at room temperature with 5% non-fat milk in TBS-T and incubated overnight at 4 °C with primary antibodies (Yap, Proteintech; β-catenin, BD Biosciences; β-actin, Santa Cruz) in 2.5% non-fat milk in TBS-T. Membranes were washed three times for 30 min in TBS-T and incubated with anti-mouse and anti-rabbit IgG 488 AlexaFluor antibodies (ThermoFisher) for 1 h at room temperature in 2.5% non-fat milk in TBS-T and then washed three times again. Finally, membranes were imaged on a Chemidoc MP (Bio-Rad).

### Computational modelling

Cavity expansion and its effect on cell density were simulated using a custom two-dimensional (2D) particle-based model. At each time step, the pairwise distances between all cells are computed and converted into forces based on an anharmonic potential function. Cells thus strongly repulse each other at very short distances (resistance to compression), weakly attract each other at medium distances (adhesion), and do not affect each other at longer distances. The positions of cells are then updated on the basis of the summed contributions of all pairwise forces (and a small amount of Gaussian noise), scaled by the size of the time step (Δ*t*). The model was initialized as a circular band of uniformly spaced cells, with the outer-most ring of cells being fixed in place to represent the stiff vitelline membrane around the embryo. The cavity was modelled as a single large cell at the centre that only imposes repulsive forces and whose rest size was gradually increased over time to reflect the expansion of the cavity. The model was implemented in Python 3.9.7 (Python Software Foundation), and the complete code is on GitHub and can be accessed under ‘Code availability’ section.

### Statistics

Data were tested for normality using the d’Agostino-Pearson and/or Shapiro–Wilk test using Prism9 (GraphPad). For two group comparisons, significances were calculated with Student’s *t*-test (two-tailed, unpaired) for data that passed the normality test or Mann–Whitney (two-tailed, unpaired) for data that did not pass the normality test. For more than two groups, comparison one-way analysis of variance (ANOVA) with Dunnett’s correction test for data that passed the normality test and Dunn’s correction test for data sets that did not pass the normality test. All statistics tests were done at 95% confidence intervals (CIs). No statistical tests were performed to determine the sample size; it was based on previous studies in the field. Each experiment was repeated at least three times on different days and with different batches of embryos. The authors were not blinded during the experiment (embryos were selected on the basis of viability or exclusion criteria mentioned in Methods sections of ‘Hydrostatic pressure measurement’ and ‘Graft and in vitro neural crest experiments’). After selecting embryos, analysis was done at random.

### Reporting summary

Further information on research design is available in the Nature Portfolio Reporting Summary linked to this article.

## Online content

Any methods, additional references, Nature Portfolio reporting summaries, source data, extended data, supplementary information, acknowledgements, peer review information; details of author contributions and competing interests; and statements of data and code availability are available at https://doi.org/10.1038/s41556-024-01378-y.

## Supplementary information

Supplementary Information Supplementary Fig. 1 and its legend.

Reporting Summary

## Source data

Source Data Figs. 1–7 and Extended Data Figs. 1–10 Statistical source data.

## Data Availability

Source data are provided with this paper. All other data supporting the findings of this study are available within the manuscript or can be obtained from the corresponding author on reasonable request.

## Code availability

The custom 2D particle-based model was implemented in Python 3.9.7 (Python Software Foundation) and available from GitHub at https://github.com/WhoIsJack/cavity_expansion.
