# Supplementary material for: Competence for neural crest induction is controlled by hydrostatic pressure through Yap
Source: Nat Cell Biol. 2024 Mar 18;26(4):530–41. doi: 10.1038/s41556-024-01378-y (PMC11021196; doi:10.1038/s41556-024-01378-y)
Supplement: Supplementary file 1 — Supplementary Fig. 1 and its legend. [file 41556_2024_1378_MOESM1_ESM.pdf]

nature cell biology

Article

https://doi.org/10.1038/s41556-024-01378-y

Competence for neural crest induction is controlled by hydrostatic pressure through Yap

In the format provided by the authors and unedited

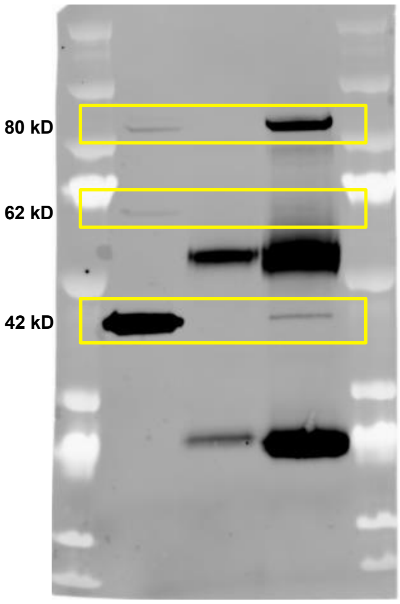

Supplementary 1 | Yap and B-catenin interaction in induced neural crest cells. Co-immunoprecipitation of induced neural crest cell lysate. Left side bands indicate input, and right side indicates positive band.
