# Supplementary material for: Competence for neural crest induction is controlled by hydrostatic pressure through Yap
Source: Nat Cell Biol. 2024 Mar 18;26(4):530–41. doi: 10.1038/s41556-024-01378-y (PMC11021196; doi:10.1038/s41556-024-01378-y)
Supplement: Supplementary file 2 — Reporting Summary [file 41556_2024_1378_MOESM2_ESM.pdf]

nature portfolio

Corresponding author(s): Roberto Mayor

Last updated by author(s): 28-01-2024

Reporting Summary

Nature Portfolio wishes to improve the reproducibility of the work that we publish. This form provides structure for consistency and transparency in reporting. For further information on Nature Portfolio policies, see our Editorial Policies and the Editorial Policy Checklist.

Statistics

For all statistical analyses, confirm that the following items are present in the figure legend, table legend, main text, or Methods section.

n/a	Confirmed	
☐	☒	The exact sample size (*n*) for each experimental group/condition, given as a discrete number and unit of measurement
☒	☐	A statement on whether measurements were taken from distinct samples or whether the same sample was measured repeatedly
☐	☒	The statistical test(s) used AND whether they are one- or two-sided<br>*Only common tests should be described solely by name; describe more complex techniques in the Methods section.*
☒	☐	A description of all covariates tested
☐	☒	A description of any assumptions or corrections, such as tests of normality and adjustment for multiple comparisons
☐	☒	A full description of the statistical parameters including central tendency (e.g. means) or other basic estimates (e.g. regression coefficient) AND variation (e.g. standard deviation) or associated estimates of uncertainty (e.g. confidence intervals)
☐	☒	For null hypothesis testing, the test statistic (e.g. *F*, *t*, *r*) with confidence intervals, effect sizes, degrees of freedom and *P* value noted<br>*Give P values as exact values whenever suitable.*
☒	☐	For Bayesian analysis, information on the choice of priors and Markov chain Monte Carlo settings
☒	☐	For hierarchical and complex designs, identification of the appropriate level for tests and full reporting of outcomes
☐	☒	Estimates of effect sizes (e.g. Cohen's *d*, Pearson's *r*), indicating how they were calculated

Our web collection on statistics for biologists contains articles on many of the points above.

Software and code

Policy information about availability of computer code

Data collection	Data was collected using Microsoft Office Excel (360) and ImageJ 2.14. Custom code availability: https://github.com/WhoIsJack/cavity_expansion.
Data analysis	Data was analyzed using GraphPad Prism 9 for statistics and graph design. InkScape1.2 for production of figures. Python 3.9.7 used to generate custom code.

For manuscripts utilizing custom algorithms or software that are central to the research but not yet described in published literature, software must be made available to editors and reviewers. We strongly encourage code deposition in a community repository (e.g. GitHub). See the Nature Portfolio guidelines for submitting code & software for further information.

Data

Policy information about availability of data

All manuscripts must include a data availability statement. This statement should provide the following information, where applicable:

- Accession codes, unique identifiers, or web links for publicly available datasets
- A description of any restrictions on data availability
- For clinical datasets or third party data, please ensure that the statement adheres to our policy

Source data are provided with this study. All other data supporting the findings of this study are available within manuscript or from the corresponding author on reasonable request.

Research involving human participants, their data, or biological material

Policy information about studies with human participants or human data. See also policy information about sex, gender (identity/presentation), and sexual orientation and race, ethnicity and racism.

Reporting on sex and gender	N/A
Reporting on race, ethnicity, or other socially relevant groupings	N/A
Population characteristics	N/A
Recruitment	N/A
Ethics oversight	N/A

Note that full information on the approval of the study protocol must also be provided in the manuscript.

Field-specific reporting

Please select the one below that is the best fit for your research. If you are not sure, read the appropriate sections before making your selection.

☒ Life sciences ☐ Behavioural & social sciences ☐ Ecological, evolutionary & environmental sciences

For a reference copy of the document with all sections, see nature.com/documents/nr-reporting-summary-flat.pdf

Life sciences study design

All studies must disclose on these points even when the disclosure is negative.

Sample size	No statistical method used to determine sample size. It was determined based on previous reports (i.e., Barriga et al., 2018, Nature; Barriga et al 2013, JCB; Alvizi L et al., 2023; Shellard et al., 2021, Nature, etc.)
Data exclusions	Any excluded date were explained fully in the method section within the manuscript. Briefly, for pressure measurement, embryos showed rapid decrease in pressure and infused ectopic expression were excluded.
Replication	Each experiment was replicated at least three times. Each replicate consisted of a different patches of samples (embryos) on different days.
Randomization	Parameters for each experiment were allocated to experimental groups and measured at random. Specified in methods section in manuscrupit.
Blinding	Authors were not blinded because embryos prior analysis and the criteria for selection was based on survival and the exclusion carteria as stated in section of the methods.

Reporting for specific materials, systems and methods

We require information from authors about some types of materials, experimental systems and methods used in many studies. Here, indicate whether each material, system or method listed is relevant to your study. If you are not sure if a list item applies to your research, read the appropriate section before selecting a response.

Materials & experimental systems

n/a	Involved in the study	
☐	☒	Antibodies
☐	☒	Eukaryotic cell lines
☒	☐	Palaeontology and archaeology
☐	☒	Animals and other organisms
☒	☐	Clinical data
☒	☐	Dual use research of concern
☒	☐	Plants

Methods

n/a	Involved in the study	
☒	☐	ChIP-seq
☒	☐	Flow cytometry
☒	☐	MRI-based neuroimaging

Antibodies

Antibodies used	Yap1 Polyclonal antibody (Proteintech, 13584-1-AP). - [1/100 or 200].

Antibodies used	Anti-β-Catenin antibody (BD Transduction Laboratories, 610153). - [1/100 or 200].<br>Anti-Sox9 (Sigma, HPA001758) - [1/500].<br>anti-Sox10 (DSHB, AB_2722342) - [1/50].<br>Anti-Digoxigenin-AP Fab fragments (Roche, 11093274910) - [1/1000].<br>goat anti-mouse Alexa Fluor 488 (Thermo Fisher Scientific, A-11001) - [1/350].<br>goat anti-rabbit Alexa Fluor 555 (Thermo Fisher Scientific, A-21429) - [1/350].
Validation	Yap1 Rabbit Polyclonal antibody (Proteintech, 13584-1-AP) - [IF Mariaceleste Aragona et al., 2020, Nature; Ievgenia Pastushenko et al., 2021, Nature].<br>Anti-β-Catenin antibody (BD Transduction Laboratories, 610153). - [IF: Eger A et al., 2000 JCB; Fallone F et al., 2013, Oncogene].<br>Anti-Sox9 (Sigma, HPA001758) - [IF: Qi Sun et al., 2017, Oncotarget].<br>anti-Sox10 (DSHB, AB_2722342) - [IF: Alvizi L et al., 2023 Nature communication].<br>Secondary antibodies were commercially validated and have been widely used in Xenopus laevis (i.e., Barriga et al., 2018, Nature; Barriga et al 2013, JCB; Shellard et al., 2021, Nature, etc):<br>Anti-Digoxigenin-AP Fab fragments (Roche, 11093274910) - [1/1000].<br>goat anti-mouse Alexa Fluor 488 (Thermo Fisher Scientific, A-11001) - [1/350].<br>goat anti-rabbit Alexa Fluor 555 (Thermo Fisher Scientific, A-21429) - [1/350].

Eukaryotic cell lines

Policy information about cell lines and Sex and Gender in Research

Cell line source(s)	iPSCs - NIBSC8 (National Institute for Biological Standards and Control - UK).
Authentication	None was authenticated.
Mycoplasma contamination	Negative result for mycoplasma contamination obtained by preforming the test with MycoAlert from Lonza.
Commonly misidentified lines<br>(See ICLAC register)	No commonly misidentified cell lines per ICLAC register were used.

Animals and other research organisms

Policy information about studies involving animals; ARRIVE guidelines recommended for reporting animal research, and Sex and Gender in Research

Laboratory animals	Adult female Xenopus laevis were used to obtain oocytes, which were fertilized with male sprem. Xenopus were age 2-5 years old. Xenopus embryos in this study were at neurula stages.<br>CD1 Female (age of 3-8 months musculus, RT) were euthanized with CO2 and embryos were collected .
Wild animals	No wild animals were used in this study
Reporting on sex	Embryo of Xenopus and mice were obtained from females. Sex was not included in experimental design as the focus of this study is within early embryonic development. Thus, to our best knowledge, our data does not apply sex-based phenotypes - no sex-based information was collected.
Field-collected samples	No feild-collected samples were used in this study
Ethics oversight	Animal licenses were approved by the Animal Welfare and Ethical Review Board (WERB) at University College London and issued by the Home Office, UK.

Note that full information on the approval of the study protocol must also be provided in the manuscript.

Plants

Seed stocks	N/A
Novel plant genotypes	N/A
Authentication	N/A
